# Nutrition Education to Reduce Metabolic Dysfunction for Spinal Cord Injury: A Module-Based Nutrition Education Guide for Healthcare Providers and Consumers

**DOI:** 10.3390/jpm12122029

**Published:** 2022-12-07

**Authors:** Alicia Sneij, Gary J. Farkas, Marisa Renee Carino Mason, David R. Gater

**Affiliations:** 1 Department of Physical Medicine and Rehabilitation, University of Miami Miller School of Medicine, P.O. Box 016960 (C-206), Miami, FL 33101, USA; 2 Christine E. Lynn Rehabilitation Center for the Miami Project to Cure Paralysis, Miami, FL 33101, USA; 3 The Miami Project to Cure Paralysis, University of Miami Miller School of Medicine, Miami, FL 33136, USA; 4 South Florida Spinal Cord Injury Model System, University of Miami Miller School of Medicine, Miami, FL 33101, USA

**Keywords:** nutrition education, spinal cord injury, cardiometabolic health, neurogenic obesity, energy balance

## Abstract

Spinal cord injury (SCI) results in a high prevalence of neurogenic obesity and metabolic dysfunction. The increased risk for neurogenic obesity and metabolic dysfunction is mainly due to the loss of energy balance because of significantly reduced energy expenditure following SCI. Consequently, excessive energy intake (positive energy balance) leads to adipose tissue accumulation at a rapid rate, resulting in neurogenic obesity, systemic inflammation, and metabolic dysfunction. The purpose of this article is to review the existing literature on nutrition, dietary intake, and nutrition education in persons with SCI as it relates to metabolic dysfunction. The review will highlight the poor dietary intakes of persons with SCI according to authoritative guidelines and the need for nutrition education for health care professionals and consumers. Nutrition education topics are presented in a module-based format with supporting literature. The authors emphasize the role of a diet consisting of low-energy, nutrient-dense, anti-inflammatory foods consistent with the Dietary Guidelines for Americans’ MyPlate to effectively achieve energy balance and reduce the risk for neurogenic obesity and metabolic dysfunction in individuals with SCI.

## 1. Introduction

The spinal cord allows for continuous communication between the brain and the rest of the body, providing vital sensory and motor information necessary for sensation, movement, reflexes, and autonomic function [1]. However, a spinal cord injury (SCI) from trauma or disease of the spinal cord disrupts the impulse conduction through the cord, which can lead to temporary or permanent paraplegia (paralysis of the lower extremities) or tetraplegia (paralysis of all four extremities), with devastating physiological consequences [2]. Due to paralysis, the injury to the spinal cord directly impacts mobility, activities of daily living, and social life. In addition, SCI results in respiratory dysfunction, cardiovascular dysfunction, cardiometabolic syndrome, neuropathic pain, spasticity, neurogenic bladder, neurogenic bowel, pressure injuries, bone metabolism dysfunction, osteopenia/osteoporosis, sexual dysfunction, infertility, and psychosocial dysfunction [1,3,4,5,6,7,8].

This manuscript primarily focuses on the impact of nutrition on comorbidities following SCI, including neurogenic obesity, neurogenic bowel, pressure injuries, osteoporosis, and cardiometabolic disease. The review briefly addresses the nutritional health status of persons with SCI and the need for nutrition education to mitigate risk for the aforementioned conditions. Module-based nutrition education is provided, which highlights the topics of nutrition that should be addressed by both health care professionals and consumers to mitigate risks of SCI- and diet-related co-morbidities.

## 2. Pathophysiology of SCI

During the acute phase of SCI, spinal shock occurs, which results in the loss of sympathetic innervation and motor function [2]. The individual is usually on mechanical ventilation as respiratory muscles are often inactive [2]. During this time, paralyzed muscle atrophies rapidly (obligatory sarcopenia), until the protein reservoir of skeletal muscle [9] and bone [10,11] is reduced to a minimum level [2]. Consequently, the body undergoes substantial physiological remodeling during the acute and subacute phases of SCI, which significantly affects the basal/resting metabolism and subsequently energy requirements, thus re-establishing metabolic homeostasis and shifting energy balance [2,12].

## 3. Energy Balance

Energy balance refers to the dynamic relationship between energy intake and energy expenditure [12]. Energy balance (i.e., metabolic homeostasis) is achieved when energy expenditure equally offsets energy intake [12]. Total daily energy expenditure (TDEE) is the sum of the basal metabolic rate (BMR) and the thermic effects of food digestion (TEF) and physical activity (TEPA) [12,13]. BMR is the minimum energy required to sustain life and contributes the most, approximately 60–70% of the TDEE [12,13]. The TEF is the energy needed for digestion and absorption after consuming food while TEPA is the energy required to sustain activities performed [12].

Food provides energy in the form of calories, and when written with a capital C, it represents kilocalories (kcals) [12]. Food provides energy from macronutrients including carbohydrates, protein, fats, and alcohol (Table 1). Carbohydrates and protein provide four kcals/gram, fat is an energy-dense macronutrient providing nine kcals/gram, and alcohol provides seven kcals/gram [12,13]. Micronutrients (i.e., vitamins and minerals) do not provide energy in the form of calories (Table 2); however, they are needed for proper functioning such as cellular communication, nutrient transport, wound healing, bone remodeling, and acid-base balance [2].

Recommended daily allowances (RDA) of macronutrients and micronutrients are frequently reviewed and updated by respected authorities such as the US Department of Agriculture (USDA) Dietary Guidelines for Americans (DGA) [18], Institute of Medicine Dietary Reference Intakes [21], and several international guidelines. While critical for good health, these guidelines are intended for the population without SCI to meet nutritional requirements [2]. However, several dietary requirements are affected in the context of SCI because of the physiological adjustments that occur to establish metabolic homeostasis, which will be discussed throughout the article.

## 4. Neurogenic Obesity

Obesity is the accumulation of excess adipose tissue associated with cardiovascular disease risk with body fat percentages >22% in men and >35% in women [12]. The prevalence of obesity in persons with SCI has been reported as high as 97% when using body fat percentages instead of body mass index to account for shifts in body composition [12]. To ensure that skeletal muscle is maintained and sarcopenia is avoided, fat mass and lean body mass should be regularly assessed.

The term *neurogenic obesity* refers to obesity that is directly linked to neurological impairment, including obligatory sarcopenia, neurogenic osteoporosis, neurogenic anabolic deficiency, sympathetic dysfunction, and blunted satiety characterized in SCI, which affects energy balance [22]. Due to the loss of metabolically active skeletal muscle (30–60% of lean body mass) [23], the BMR of persons with SCI is 12–54% lower than those without SCI [2,24,25]. In addition, the TEPA is also reduced due to the loss of skeletal muscle, resulting in an even further reduction in energy expenditure [12]. TEF is also notably reduced after SCI [26,27], thereby affecting TDEE. The significant reduction of BMR, TEPA, and TEF yields a markedly reduced TDEE due to the abovementioned factors. Therefore, energy intake in the form of foodstuffs should be adjusted accordingly to maintain energy balance [26,28,29].

To avoid overfeeding, an accurate determination of energy needs is required. Predicted energy expenditure equations used in the population without SCI to determine energy and nitrogen needs (protein) overestimate actual energy needs by >25% in persons with SCI [2,12,25,29]. In the population without SCI, energy needs are estimated to be 25–35 kcals/day in females and 30–40 kcals/day in males [30]. However, energy needs estimations must be adjusted for the population with SCI who exhibit lower energy expenditure. Cox et al. [31] demonstrated energy intake parameters of 27.9 kcals/kg/d for persons with paraplegia and 22.7 kcals/kg/d for persons with tetraplegia using BMR in the Weir equation [32] of 1949. Unfortunately, this study has not been replicated, nor did it consider other factors that could affect TDEE, such as spasticity, wounds, hormones, and autonomic dysreflexia [12,31]. Nonetheless, the Academy of Nutrition and Dietetics (AND) adopted these energy intake parameters in their 2009 SCI Evidence-Based Nutrition Practice Guidelines [30] and has been used in the field since.

Ideally, energy needs should be estimated with BMR or resting metabolic rate (RMR) via indirect calorimetry and applying a physical activity factor level of 1.25–1.75 in persons without SCI [30]. In the population with SCI, Farkas et al. [2] recommend assessing B/RMR in persons with SCI via indirect calorimetry; however, if unavailable, B/RMR can be calculated using SCI-specific prediction equations developed by Nightingale and Gorgey [33], Chun et al. [34] or Buchholz et al. [35]. TDEE and subsequently energy needs can be determined with the prediction equations and the Farkas et al. [36] SCI-specific correction factor of 1.15.

## 5. Weight/Adipose Tissue Management in SCI

To minimize the adverse physiological effects secondary to SCI, a heart-healthy dietary pattern that is low-calorie yet nutrient-dense is recommended to promote a good body habitus [13,37]. As nutrition plays a significant role in health promotion and disease prevention and management, clinicians are expected to educate patients on nutrition; however, there is a lack of nutrition knowledge in these professionals and/or confidence in disseminating nutrition education [38,39]. Pellegrini et al. [40] reported that common barriers to counseling for weight management amongst SCI healthcare providers included individual-level factors, such as the healthcare providers’ lack of knowledge and poor dietary strategies [40]. In support of this, a study by Burkhart et al. [41] indicated that provision of education and encouragement of healthy behaviors by health care providers were effective methods for weight management among persons with SCI.

Lavela et al. [42] reported that individuals with SCI desired information on tailored diets specifically for individuals with paraplegia and tetraplegia, including examples of healthy foods that are accessible and easily prepared by persons with SCI, which is what Farkas and colleagues [2] reported in their recent review. Altogether, the findings on weight management have a common theme of providing tailored education to increase knowledge and promote healthy behavior in individuals with SCI; however, there is a need for the standardization of the best practices guidelines offered to persons with SCI who have unique nutritional requirements.

## 6. SCI-Specific Nutritional Guidelines

There have been limited nutritional guidelines for the population with SCI; however, recent, and more explicit recommendations are emerging. The AND released an SCI Evidence-Based Nutrition Practice Guidelines [30] over a decade ago, which provided guidance for energy and protein intake in both the acute and chronic phases of SCI. Since then, the Clinical Practice Guidelines on the Identification and Management of Cardiometabolic Risk After SCI from the Paralyzed Veterans of America (PVA) have provided the first nutritional recommendations for persons with SCI [28,43]. A more recent publication by Farkas et al. [2] provided a comprehensive review and practical nutritional recommendations for persons with SCI.

## 7. Overview of the Recent SCI-Specific Nutritional Recommendations

Recommendations from the PVA Clinical Practice Guidelines on the Identification and Management of Cardiometabolic Risk After SCI [43] include adopting a heart-healthy nutrition plan, implementing the Dietary Approach to Stopping Hypertension diet or Mediterranean-based cuisine if cardiometabolic risk factors are present, limiting saturated fat intake to 5–6% of total caloric intake, and limiting sodium intake to ≤2400 mg for individuals with hypertension [43]. The PVA Clinical Practice Guidelines [43] further expand on the heart-healthy diet to include fruits, vegetables, whole grains, low-fat dairy, poultry, fish, legumes, non-tropical vegetable oils, and nuts while limiting sweets, sugar-sweetened beverages, and red meat. These guidelines were the first developed to improve the nutritional habits of persons with SCI, but more specific recommendations were needed.

Farkas et al. [2] expanded on the PVA Clinical Practice Guidelines in a recent review and provided recommendations that included specific instructions for portion sizes, frequency of consumption, and food variations. Such suggestions included that persons with SCI should consume lean poultry in moderate amounts of three-to-four-ounce portions and should eat fish two times per week [2]. The authors [2] emphasize that all persons with SCI, regardless of hypertension status, should adopt a diet with ≤2400 mg of sodium per day because the literature consistently reports that salt consumption is elevated in the diet of individuals with SCI. Fruit intake of two to three servings per day should be from whole fruits rather than juices due to differences in sugar and fiber content. Vegetable intake is also emphasized at three to four servings a day, consisting of the five vegetable subgroups (dark green, red and orange, legumes, starchy, and other vegetables) [2,18]. Dairy products are encouraged in the forms of low-fat milk, yogurt, and cheese, while limiting saturated fat intake to 5–6% of daily calories [2]. Sugar-sweetened beverages should be replaced with zero-calorie water; high-fat, sugary sweets should be replaced with fresh fruits [2]. Red meat and sweets should be consumed sparingly and on special occasions [2].

The authors also recommend a reduced emphasis on restricting dietary macronutrients in persons with SCI and focusing on consuming a healthy dietary pattern [2]. This emphasis on establishing healthy dietary habits rather than focusing on individual nutrient intake is also promoted in the 2020–2025 Dietary Guidelines for Americans (DGA) [18]. Persons with SCI who follow a healthy diet will naturally consume higher amounts of beneficial nutrition components and lower amounts of unhealthy nutrition components [2]. Moreover, while supplementation is useful to correct any identified deficiencies, obtaining nutrients via a healthy diet in lieu of supplementation is recommended to ensure a complete panel of nutrition and prevent toxicity associated with high doses [2].

Lastly, other studies have demonstrated positive cardiometabolic health benefits via a reduced calorie diet. The Diabetes Prevention Program [44] is a highly successful lifestyle intervention aimed to reduce diabetes risk and incidence (a cardiovascular risk factor) in the general population. The intervention was administered to 1079 participants without SCI who were encouraged to lose seven percent of their body weight via exercise and a low-calorie diet, and resulted in a notable 58% reduction in the incidence of diabetes [44]. Among the SCI-population, Bigford et al. [45] recommended the adaptation of the Diabetes Prevention Program to similarly yield cardiometabolic benefits through weight loss via calorie restriction.

## 8. Nutrition Education Modules

Nutrition education specific to persons with SCI is still in its infancy. However, the following modules (see Supplementary Materials), based on the latest evidence-based literature are put forth as a foundation for nutrition education targeted for persons with SCI and health care professionals. Achieving optimal health is a multidisciplinary task, and while non-dietetic health care providers can initiate primary nutrition education, follow-up with medical nutrition therapy (consisting of in-depth education and counseling) provided by registered dietitians is necessary to ensure long-term health and well-being. While these modules may serve as a foundation for nutrition education, health care professionals from different disciplines (e.g., physicians, exercise specialists, dietitians, etc.) must consider other comorbidities (e.g., Crohn’s disease, allergies, irritable bowel syndrome, etc.) and current TDEE before prescribing a specific diet. Tailoring these education modules to individual’s health status, preferences, and resources is important for optimizing adherence and health outcomes.

### 8.1. Module 1—MyPlate: A Guide to Healthy Eating

MyPlate is the consumer guide based on the 2020–2025 DGA intended to serve as a model of healthy meals for Americans [46]. The key recommendations from the 2020–2025 DGA focus on the five MyPlate Groups (Fruit, Vegetable, Grain, Protein, and Dairy Groups) which are explained in Modules 2 through 7. MyPlate optimizes nutritionally dense foods with half the plate comprised of fruits and vegetables, approximately a quarter of the plate with grains, slightly less than a quarter of the plate with protein, and including a serving of dairy in every meal [18,46].

The Healthy Eating Index (HEI) is a tool to measure diet quality and assess how well it aligns with MyPlate, based on the major recommendations from the DGA [47]. The HEI is calculated by ten components based on the DGA guidelines, worth ten points each with a maximum score of 100 [47]. Both persons with and without SCI have been shown to generally have low HEI scores, with a reported average score of 59 for persons without SCI [18] and scores ranging from 47–60 for persons with SCI [48,49,50]. In persons with SCI, Li et al. [48] reported an HEI score of 47 based on the 2015–2020 DGA, a value that is ten units lower than the HEI reported by the USDA for the population without SCI [18]. Tomey et al. [49] evaluated the dietary intake and nutrition knowledge of 95 community-dwelling men with paraplegia and compared those findings with data collected from the 1999–2000 National Health and Nutrition Examination Survey. Based on the 2000–2005 DGA, the authors [49] reported an HEI score of 59.8 for men with paraplegia and 61.3 for men without SCI. Silveira et al. [50] assessed the quality of 37 persons with chronic SCI using the Automated Self-Administered 24 h Recall dietary assessment for three non-consecutive days. The authors [50] reported an HEI score of 54.4 based on the 2015–2020 DGA. Collectively, the overall diet quality based on the DGA for persons with SCI has been reported to be similar or worse to those without SCI, indicating the need for improvement.

The association between the HEI score and cardiovascular risk factors has been assessed after SCI. Although limited, evidence is emerging that associate improvements in diet quality reflecting the DGA recommendations with better cardiometabolic profiles. In 24 persons with chronic SCI, Li et al. [48] demonstrated that higher conformance to the DGA, as assessed by the HEI, was moderately associated with a better fasting glucose profile and trivially associated with glucose metabolism, blood lipids, and C-reactive protein. In another article by Li et al. [51], the authors observed that a 3.3 mg/dL reduction in fasting glucose was associated with a 10-point increase in HEI scores. The authors [51] noted improved cardiovascular risk factors for total cholesterol, high-density lipoproteins cholesterol, and low-density lipoprotein cholesterol with higher HEI scores in persons with SCI.

The importance of Module 1 in introducing general knowledge of dietary patterns, and specifically the MyPlate/DGA recommendations, to individuals with SCI is supported in the literature. Tomey et al. [49] observed a significant positive correlation between nutrition knowledge and diet quality in men with paraplegia, indicating that low nutrition literacy increases the risk for poor diet quality. Research evaluating the effectiveness of MyPlate has identified the need for interventions to increase MyPlate public awareness and improve diet quality through the adoption of the DGA among Americans [52], including persons with SCI.

Collectively, the above overview of findings demonstrates poor diet quality among persons with SCI and the relationship among a suboptimal diet, poor nutrition knowledge, and cardiovascular risk. Thus, there is an indicated need for enhanced nutrition education in individuals with SCI.

### 8.2. Modules 2 and 3—The Fruit and Vegetable Groups

#### 8.2.1. The Fruit Group

The Fruit Group consists of whole fruits and 100% fruit juice, which can be fresh, canned, frozen, or dried. Examples of fruit and cup equivalents can be found in Table 3 [18]. Although fruit juice is included in the Fruit Group, it should be 100% fruit juice without added sugars and additives [18]. Of note, fruit drinks are not to be confused with fruit juices. Fruit drinks often do not contain any fruit juice but are typically flavored after fruit and are energy-dense with more than 200 calories and approximately 60 g of added sugar per 12-ounce serving [53]. Consequently, fruit drinks do not count toward the fruit intake guidelines.

The 2020–2025 DGA further recommends that more than half of fruit intake be derived from whole fruit instead of juice, which is depleted of fiber [18]. While fresh fruit is ideal, frozen fruit is a convenient and cost-effective way of meeting fruit recommendations as it is flash-frozen immediately upon harvest, preserving nutrient content [18]. Canned fruits can be consumed, especially when they are not always found fresh in the grocery store year-round (Table 3) [18]. As a form of whole fruit, dried fruit without added sugar contains fiber and is a good source of vitamins A and C [18].

#### 8.2.2. The Vegetable Group

The Vegetable Group is divided into five subgroups and includes: (1) dark green vegetables; (2) red and orange vegetables; (3) beans, peas, and lentils; (4) starchy vegetables; and (5) other vegetables [18]. Green peas and green/string beans are not included in the beans, peas, and lentils subgroup because their nutritional profile is more comparable to vegetables of other subgroups [18]. Table 4 lists examples of vegetables that fall under each subcategory and their recommended intakes. While fresh vegetables are best, vegetables can also be frozen, canned, dried/dehydrated, and consumed raw, cooked, or juiced.

#### 8.2.3. Current Fruit and Vegetable Intake

Fruit and vegetable intake recommendations from the 2020–2025 DGA include 2 and 2.5 cup equivalents per day, respectively [18]. However, 80% of the US population does not meet current recommendations for fruit, while 90% do not meet vegetable recommendations [18]. Likewise, individuals with SCI do not meet the DGA’s recommendation for fruit and vegetable intake [50,54,55]. Silveira et al. [50] reported inadequate fruit intake among persons with chronic SCI with an average daily intake of slightly more than half a cup, roughly one and a half cups lower than 2015–2020 DGA recommendations. The same authors [50] also noted that vegetable consumption did not meet DGA recommendations, with an average intake of fewer than two cups per day. Lieberman et al. [55] observed a significantly lower intake of fruit at 2.4 servings per day (adjusted for total energy intake) in individuals with chronic SCI (n = 100) compared to 3.9 servings per day in age, sex, and race-matched controls (n = 100). In addition, the authors [54] identified lower consumption of vegetables in persons with SCI (4.10 servings per day) compared to individuals without SCI (4.93 servings per day), but these findings were not significant.

#### 8.2.4. Health Benefits of Fruits and Vegetables

In a meta-analysis by Aune and colleagues [56], the authors reviewed the literature for populations without SCI and assessed the strength and shape of the dose–response relationship between the intake of fruits and vegetables on several morbidities. The authors [56] demonstrated for the intake of fruits and vegetables combined, a relative risk per 200 g/day was 0.92 for coronary heart disease, 0.84 for stroke, 0.92 for cardiovascular disease, 0.97 for total cancer, and 0.90 for all-cause mortality. Similar findings were reported for fruits and vegetables separately. Inverse associations were also reported between the intake of apples and pears, citrus fruits, green leafy vegetables, cruciferous vegetables, and salads and cardiovascular disease and all-cause mortality, as well as between the consumption of green-yellow and cruciferous vegetables and total cancer risk.

The cardioprotective effect of fruits and vegetables may be attributed to several of the known bioactive components, their low glycemic load, energy density (amount of energy provided in the form of calories respective to the food item’s weight [57]), and anti-inflammatory properties [58,59]. The antioxidants found in fruits and vegetable may counter reactive oxygen species and limit DNA damage [60], cruciferous vegetables contain glucosinolates which detoxify enzymes [61], and the consumption of fruits and vegetables have been reported to improve hormone levels [60]. Moreover, the consumption of fruit and vegetables has been inversely associated with body adiposity [62], potentially because fresh produce has low energy density. The low energy density and nutrient-dense profile of fruits and vegetables are especially important for persons with SCI to help achieve and maintain energy balance. MyPlate and its Fruit and Vegetable Groups provide an excellent visual of a heart-healthy diet with half the plate comprised of fruits and vegetables.

### 8.3. Module 4—The Grains Group

The Grains Group is subdivided into two categories, (1) whole-grains and (2) refined-grains [18]. Table 5 presents examples of whole-grain and refined-grain products [18]. Whole-grains are unprocessed, containing the entire kernel comprised of the bran, germ, and endosperm, which provide the fiber, micronutrient, and carbohydrate content, respectively [63]. Refined-grains are milled, which removes the bran and germ, stripping the grain from its fiber, iron, and several B vitamins (i.e., thiamin, riboflavin, niacin, and folic acid) content [18]. To compensate for nutrient depletion, refined-grains may be enriched with iron and B vitamins that were removed; however, fiber is seldom added back [18].

#### 8.3.1. Current Grain Intake

2020–2025 DGA guidelines recommend that at least 50% of total grain intake come from whole-grains [18]. Both persons with and without SCI meet or exceed total grain recommended intake; however, they do not meet recommendations for whole-grain consumption [18,29,50,54,64]. Silveira et al. [50] reported that whole-grain made up 15% of total grain intake in persons with SCI compared to 19% in controls without the injury. Lieberman et al. [54] demonstrated that individuals with SCI consumed significantly fewer servings of whole-grains (adjusted for total caloric intake) than controls without SCI. The same authors reported that only 8% of individuals with SCI met whole-grain recommendations compared to approximately 70% of controls without SCI [54]. For persons with SCI, replacing refined-grains with whole-grains is expected to provide a complete and balanced package of nutrients, without the reliance of enrichment to obtain the vitamins and minerals lost during the process of grain refinement. The nutrients from whole-grains are especially important given the population’s micronutrient deficiencies [29] and the need for a fiber-rich diet.

#### 8.3.2. Health Benefits of Whole Grain

Whole-grain consumption is associated with cardiovascular disease risk reduction [63,65]. Jacobs et al. [63] reported a 20–40% reduction of long-term risk for cardiovascular disease and other conditions in the population without SCI for those who regularly consumed whole-grains compared to those who rarely ate them. The authors [63] also identified a similar risk reduction for cardiovascular disease related to the amount of whole-grain cereal consumed. While the cardioprotective effect of whole-grain has been attributed to fiber, the authors [63] concluded that there is a synergistic effect of the different components from the whole-grain with fiber acting as one factor [63]. Similarly, Aune et al. [65] in a meta-analysis with general population studies demonstrated an association between whole-grain intake and reduced risk for coronary heart disease, cardiovascular disease, total cancer, and all-cause mortality. Collectively, these results confirm the need for whole-grain consumption for optimal health and given the decreased intake of whole-grains in persons with SCI compared to the general population, this need is even more significant.

### 8.4. Modules 5 and 6—The Protein Group

Protein is found in both animal- and plant-based sources and is divided into three subcategories: (1) meats, poultry, and eggs; (2) seafood; and (3) nuts, seeds, and soy products [18]. Table 6 presents the 2020–2025 DGA recommendations for protein intake and examples. Protein is not exclusively found in the Protein Group but also in other MyPlate Groups. Because of its high protein content, food in the Dairy Group can also satisfy the dietary requirements of the Protein Group, but not in the same meal. A similar situation applies to legumes (i.e., beans, peas, etc.) as they have a high protein content and can count towards the dietary requirements of the Protein or the Vegetable Group, but not both. Of note, green peas or string/green beans are not in the Protein Group because these vegetables have a low protein content compared to other legumes [18].

#### 8.4.1. Amino Acids

Amino acids found in protein are the building blocks for protein synthesis and are categorized as essential, non-essential, and conditionally essential amino acids (Table 1). Of the nine essential amino acids, leucine, isoleucine, and valine are branched-chain amino acids and are associated with muscle protein synthesis [66]. Protein should be consumed from all subgroups to ensure adequate ingestion of amino acids, especially essential amino acids that cannot be synthesized.

In persons with SCI, variability and inadequacy of certain amino acid intake have been reported. Groah et al. [67] observed that amino acid intake in 73 persons with chronic SCI met or approached the RDA, except for threonine, methionine, lysine, leucine, and cysteine. Sabour et al. [68] reported intake of protein containing high amounts of lysine, leucine, valine, and isoleucine in 100 persons with chronic SCI. However, the authors [68] also demonstrated low intake of arginine, alanine, and aspartic acid.

#### 8.4.2. Protein Needs

The acceptable macronutrient distribution range (AMDR) for protein intake is 10–35% of total energy intake per day and the RDA is 0.8 g/kg of body weight for adults without SCI [18,69]. Similarly, according to the AND, protein recommendations for individuals with SCI range from 0.8–1.0 g/kg of body weight during the chronic phase [30]. However, for persons with SCI in the acute phase, protein requirements do not follow guidelines for the general population.

According to the AND’s SCI Nutrition Practice Guidelines [30], protein requirements increase substantially during the acute phase of the injury. The AND’s recommendation for the acute phase is 2.0 g of protein/kg of ideal body weight [30], which is more than double the general protein recommendations. The reasoning behind the increased protein requirements during the acute phase is to mitigate the negative nitrogen balance [30]; however, evidence suggests that the negative nitrogen balance during the acute phase is obligatory and cannot be prevented. Laven et al. [70] assessed the nutritional status of 51 persons with acute SCI. The authors reported that although albumin and prealbumin levels (also called transferrin), both markers of protein status, were significantly reduced during the first 8 weeks post-injury, both markers improved with time and were not associated with an increased risk of secondary medical complications. Kolpek et al. [71] compared seven patients with SCI to seven patients with head trauma for changes in urinary urea nitrogen excretion and measured energy expenditure over the first 18 days following the injury. The authors [71] reported that although urinary urea nitrogen was comparable in patients with SCI and head trauma, there was a significant difference in the ratio between measured energy expenditure and predicted energy expenditure between the groups. This finding suggests that the elevation of urinary urea nitrogen observed in SCI is not due to a hypermetabolic state but is a result of other mechanisms related to disuse and denervation atrophy [71]. Rodriguez et al. [72] compared ten patients with acute SCI to 20 controls with non-spinal cord injuries matched for time since injury, sex, age, and injury severity score. The authors [72] reported that no SCI patient established positive nitrogen balance during the seven-week period following injury despite an average delivery of 2.4 g of protein/kg of ideal body weight and 120% of the predicted energy expenditure at the time of peak negative nitrogen balance. However, 17 of the 20 non-SCI patients achieved positive nitrogen balance within three weeks of admission [72]. The authors [72] concluded that their findings demonstrate the phenomenon of obligatory negative nitrogen balance acutely following SCI and that aggressive attempts to achieve positive nitrogen balance will ultimately fail and result in overfeeding. In a 2011 systematic review on the acute management of nutritional demands after SCI, Thibault-Halman et al. [73] reported a reduction in metabolic activity during the acute phase, along with an unpreventable negative nitrogen balance despite aggressive nutritional supplementation. The authors [73] concluded that nutritional support provided during the acute phase must be accurately monitored to avoid potential overfeeding. Shin et al. [74] demonstrated increased fat mass during acute rehabilitation, which was negatively correlated with functional improvement. This practice of overfeeding patients during the acute phase sets the stage for the accumulation of adipose tissue.

In the presence of a pressure injury, The 2014 National Pressure Ulcer Advisory Panel, European Pressure Ulcer Advisory Panel and Pan Pacific Pressure Injury Alliance Prevention and Treatment of Pressure Ulcers Clinical Practice Guideline state that increasing protein requirements must be done with clinical judgement and must be compatible with overall goals of patient care [75]. The practice of increasing protein requirements in the presence of pressure injuries also increases energy intake if not offset with a reduction in the intake of the remaining macronutrients. Therefore, if protein intake is increased to aid with wound healing, intake of carbohydrates and/or fat must be reduced to maintain energy balance and prevent overfeeding. However, evidence suggests that additional protein may not be necessary. Cereda et al. [76] conducted a 12-week randomized controlled trial (RCT) and reported increased healing rates of pressure injuries when patients without SCI consumed a nutritional formula enriched with protein, arginine, zinc, and vitamin C (antioxidant) daily for at least eight weeks [76]. Van Anholt [77] demonstrated similar results in another RCT using the same nutritional formula used by Cereda et al. [76] in non-malnourished older patients without SCI with Stage III and IV pressure injuries. The authors [77] concluded that nutritional formula enriched with protein, arginine, and other micronutrients is associated with accelerated healing.

For persons with SCI, the AND recommends 1.2–1.5 g of protein/kg of body weight per day for Stage II and 1.5–2.0 g of protein/kg of body weight for Stages III and IV pressure injuries [30]. Similarly, the PVA’s Clinical Practice Guidelines for Pressure Ulcer Prevention and Treatment Following SCI [78] also recommend increased protein requirements ranging from 1.25–2.0 g/kg of body weight per day with the higher requirements suggested for those with injuries of greater severity.

The reasoning behind the increased protein requirements when pressure injuries are present is to compensate for the loss of lean body mass, which is essential for wound healing [24]. Chapman et al. [79] demonstrated a 2.5 faster healing rate of pressure injuries in persons with acute SCI when supplemented with additional protein containing arginine, zinc, and vitamin C, compared to those who stopped taking the supplement. Desneves and colleagues [80] in a three-week RCT compared the healing rates of pressure injuries in persons with SCI when receiving a standard hospital diet with two high protein/energy supplements or a standard diet with two high-protein/energy supplements enriched with arginine, zinc, and vitamin C. The authors [80] demonstrated a clinically significant improvement in pressure injury healing only in persons with SCI receiving a standard hospital diet plus two high protein/energy supplements enriched with arginine, zinc, and vitamin C. Brewer et al. [81] observed significantly accelerated healing time of pressure injuries in 18 persons with SCI receiving nine grams of commercial powered arginine supplement per day compared to historical controls, demonstrating the benefit of arginine supplementation on pressure injury healing without additional protein. Arginine, a conditionally essential amino acid, is highly involved in the process of wound healing; arginine assists with new blood vessel formation and tissue regeneration by increasing the release of endothelial progenitor cells from bone marrow, improves protein anabolism and cell growth, is a donor of nitric oxide which increases blood flow to the wound area and acts as an immune response mediator [82]. Collectively, these findings suggest that the supplementation of arginine, zinc, and vitamin C are driving the healing of pressure injuries and not necessarily the increased protein content.

#### 8.4.3. Current Protein Intake

Approximately three-quarters of persons without SCI consume adequate amounts of protein in the form of meat, poultry, and eggs; however, more than 50% do not meet recommendations for protein intake in the form of nuts, seeds, and soy products [18]. In addition, almost 90% of persons without SCI do not meet recommendations for protein-rich seafood [18]. Similarly, evidence indicates that protein intake among persons with SCI is within or exceeding guidelines [21,29,50,54]. Levine et al. [21] and Silveira et al. [50] observed protein intake among persons with SCI to be within the normal range; however, like the population without SCI, individuals with SCI reported inadequate intake of protein from seafood and plant-based sources [18,21,50]. Lieberman et al. [54] reported greater meat and fish/seafood consumption in persons with SCI compared to controls, though not significant. Recently, in a meta-analysis by Farkas et al. [29] the authors observed protein intake in persons with chronic SCI exceeded the 2015–2020 DGA recommendations by up to 74%; however, due to limited data, the authors were not able to provide a breakdown of the different sources of dietary protein. Collectively, these data suggest that although protein intake is within or exceeding guidelines in persons with SCI, poor quality diets may contribute to missing key essential amino acids.

Providers should correct poor quality diet by instructing individuals with SCI to occasionally consume red meat and replace highly processed, high-fat meat products (i.e., hot dogs, bacon, sausages) with seafood- and/or plant-based protein. This dietary pattern increases fiber intake, while lowering total fat, saturated fat, and salt intake. In addition to being a good source of protein, seafood is also nutrient-dense. For example, cod is abundant in protein, B vitamins (B3, B6, and B12) and several minerals (selenium, magnesium, phosphorus, and potassium), yet low in fat [83]. Three ounces of cod is equivalent to 72 calories and provides 16 g of protein (constituting 89% of total its calories) [83]. Moreover, an emphasis should be placed on consuming lean forms of protein to lower the energy density. Effective ways to lower fat content of protein products are to remove visible fat and/or skin from animal-based sources before and/or after cooking. Persons with SCI can request the supermarket butchers to trim the fat prior to purchasing. Protein products should be prepared with methods that minimize excess fat added including grilling, roasting, braising, and baking, instead of deep frying.

### 8.5. Module 7—The Dairy Group

The Dairy Group includes food products derived from milk that retain the calcium content [46]. Non-dairy products such as fortified soymilk and soy yogurt are also included in the Dairy Group because the nutritional profile is comparable to dairy milk and yogurt [46]. Although derived from milk, several milk-based products (i.e., cream, cream cheese) are excluded because they undergo processing. Table 7 provides USDA’s recommendations for dairy intake and examples according to the 2020–2025 DGA.

#### 8.5.1. Current Dairy Intake

The majority of consumed dairy products are typically high in sodium, saturated fat, and added sugar [18], nutrients eaten in high quantities in persons with and without SCI [18,49,55]. Similar to the population without SCI, inadequate intake of the Dairy Group is also observed in individuals with SCI [18,49,50,55,84]. Silveira et al. [50], Javidan et al. [84], and Tomey et al. [49] observed low dairy intake in persons with chronic SCI. Lieberman et al. [55] also observed individuals with SCI do not currently meet 2010–2015 DGA recommendations for dairy intake of 3-cup equivalents/per day for adults (age and sex-dependent). Moreover, the authors reported that individuals with SCI consumed significantly less dairy than age, sex, and race-matched controls [55]. In another study, Lieberman et al. [54] noted that only 22% of individuals with SCI met USDA’s dairy recommendations compared to 54% of controls [54].

#### 8.5.2. Dairy and Cardiovascular Disease

Studies have examined the association between dairy intake and cardiovascular health, but current results remain controversial. Dehghan et al. [85] conducted a large prospective cohort study across 21 countries in participants without SCI and found that dairy intake was associated with a lowered risk for mortality and major cardiovascular disease events. Bhupathi et al. [86] reported that most of the current evidence from randomized clinical trials and prospective trials suggest that dairy products have a neutral or protective effect on cardiovascular disease in the general population. St-Onge et al. [87] reported that fermented dairy products have a cardioprotective effect by reducing blood cholesterol levels. Although most of the fat provided by dairy sources is saturated, evidence suggests that saturated fat from dairy does not increase cardiovascular risk in persons without SCI and may even provide a slight protective effect [85,86,88]. Though the cardioprotective effect of dairy in persons with SCI has yet to be investigated, there is strong evidence to support the positive effects of dairy observed in the population without SCI.

#### 8.5.3. Dairy and Osteoporosis

More than 75% of persons without SCI do not meet the calcium intake recommendations according to the 2020–2025 DGA [88]. The current 2020–2025 DGA guidelines recommend 1000–1300 mg/day of calcium (Table 2). Several studies [50,84,89] have reported inadequate calcium intake in persons with SCI with a pooled mean of 811.1 mg/day, according to Farkas et al. [29] Doubelt et al. [89] observed that persons with SCI fell below Estimated Average Requirements of calcium intake by 13%. Tomey et al. [49] and Iyer et al. [90] reported inadequate calcium intake in persons with SCI, with low calcium intake observed in 69% of persons with SCI. Poor calcium intake is largely due to inadequate dairy intake.

Inadequate calcium intake is of concern for the population with SCI, who are at risk for osteoporosis [91,92,93]. The prevalence of osteoporosis in persons with SCI is 75% [94] compared to 18% [95] in persons without SCI. Bone serves as a reservoir for calcium and when serum calcium is low, calcium is drawn from the bones, weakening its nutritional structural integrity [96]. The 2022 PVA Clinical Practice Guidelines on Bone Health and Osteoporosis Management in Individuals with SCI [97] recommend calcium intake of 1000–1200 mg/d (sex and age-dependent), and 750–1000 mg/day for individuals with SCI and calcium oxalate stones, with a preference for dietary intake over supplements. Regarding vitamin D, the PVA Clinical Practice Guidelines [97] recommend maintenance doses of vitamin D_3_ (cholecalciferol) of 25–50 mcg/day (1000–2000 IU/day) for the SCI population, which is notably higher than the RDA for the general population of 600 IU/d.

Interestingly, Doubelt et al. [89] did not observe significant relationships between bone mineral density (a marker for osteoporosis) and intakes of calcium intake as well as vitamins D, K, and protein in persons with SCI. The same authors observed positive associations between bone mineral density and leptin, insulin, adiponectin, and visceral adipose tissue. The research by Doubelt et al. [89] suggests a link between adiposity, metabolic health, and bone composition in persons with SCI. Doherty et al. [98] reported a significant inverse association between adiponectin (an anti-inflammatory cytokine derived from adipose tissue) and bone mineral density in non-ambulatory men with SCI independent of body composition. In the same study, the authors did not find an association between adiponectin and bone mineral density in SCI men who ambulated. The authors [98] noted that for individuals with SCI, ambulating may lessen the effect of adiponectin-mediated bone loss, as the osteoprotective benefits of obesity appear to require mechanical loading while walking. Thus, this evidence suggests a link between adiposity, metabolic health, and bone composition in persons with SCI.

The PVA Clinical practice Guidelines [97] recommend that individuals with SCI, low bone mass, and moderate-to-high fracture risk be offered oral alendronate combined with adequate calcium and vitamin D3 to treat low total hip, distal femur, or proximal tibia areal bone mineral density. Fernandez et al. [99] in a one-year RCT demonstrated a significant reduction of bone mass in 26 persons with acute SCI receiving vitamin D (calcifediol) and calcium-enriched diets with alendronate (medication to prevent osteoporosis) compared to 26 persons with acute SCI receiving only vitamin D and calcium-enriched diets. However, the results from Fernandez et al. [99] could only be extrapolated to men with SCI since the random distribution of women was asymmetrical and the effect of alendronate in their bone mineral density could not be established. Nutrition education on diets abundant in calcium-rich food, vitamin D, and phosphorus are important to protect against metabolic dysfunction, osteoporosis, and risk factors for fractures in the population with SCI [100,101,102].

### 8.6. Module 8–Fat

The AMDR for total fat intake is 20–35% [14], with saturated fat limited to 10% of daily total calories [18]. However, the AHA recommends limiting saturated fat intake to no more than 5–6% and replacing it with unsaturated fats, a recommendation that the PVA’s Clinical Practice Guidelines on Identification and Management of Cardiometabolic Risk after SCI [103] and Farkas et al. [2] also support. While excessive fat intake should be avoided, fats are needed for essential physiological functions such as hormone synthesis, storage of fat-soluble vitamins, brain and nervous system maintenance, joint lubrication, skin integrity, and wound healing [104,105,106]. Table 1 presents the different forms of dietary fat, recommended intakes, and examples of sources.

#### 8.6.1. Current Fat Intake

Among persons with SCI, Levine et al. [21], Moussavi et al. [107], and Tomey et al. [49] reported an excessive intake of total fat of at least 35%; however, Levine et al. [21] reported a lower fat intake of 31.5% in females with SCI. Silveira et al. [50] found total fat intake among persons with SCI to be within guidelines; however, they were towards the upper limit of the AMDR recommendations at 34.3% ± 6.2% of total calories. In a recent meta-analysis, Farkas et al. [29] found total fat intake among persons with SCI to be within 2015–2020 DGA guidelines for men; however, the authors [29] reported that females over 31 years old had intakes of fat that exceeded DGA recommendations.

Regarding saturated fat intake, approximately 70–75% of American adults exceed the 10% limit of saturated fat recommendation [18], which is comparable to rates found in persons with SCI. Iyer et al. [90] reported that 65% of individuals with SCI exceeded the 10% limit and 100% exceeded the 5–6% limit of saturated fat intake. Tomey et al. [49] and Moussavi et al. [107] demonstrated that saturated fat intake in persons with chronic SCI exceeded the DGA’s 10% limit recommendation. Groah et al. [67] reported that saturated fat intake was greatest for males with an average intake of 11%; intake of saturated fat was observed to be at the upper limits of DGA’s recommendation for females with SCI (~10%); however, it well exceeded AHA’s recommendations.

#### 8.6.2. Health Benefits of Unsaturated Fats

Numerous studies have demonstrated the cardioprotective effects of healthy unsaturated fats, especially when saturated and trans fats are replaced. In a systematic review, Clifton et al. [108] demonstrated lowered cardiovascular disease risk when saturated fat is replaced with unsaturated fat in persons without SCI. In a recent meta-analysis of studies conducted in the general population, Zhu et al. [109] did not find intake of total fat, saturated fat, or unsaturated fat to be associated with cardiovascular disease risk; however, they observed a cardioprotective effect of polyunsaturated fat. The same authors [109] also observed a positive dose–response relationship between cardiovascular disease risk and the high intake of trans-fat. In persons with SCI, Sabour et al. [110] demonstrated that higher intakes of cholesterol and saturated fat were associated with increased blood pressure (a cardiovascular risk factor), whereas intake of polyunsaturated fat (docosahexaenoic acid) had a blood pressure-reducing effect. Myers et al. [111] observed improved plasma insulin, homeostatic model assessment insulin resistance, and total cholesterol/high-density lipoprotein ratio after a 24-month cardiovascular risk reduction program in 26 males with SCI; the authors [111] reported a reduction in saturated fat intake with no change in total fat, though the reduction was not significant. Thus, given the benefit of unsaturated fat, health care providers should encourage their patients with SCI to reduce the consumption of unhealthy fats while increasing the intake of healthy fats. For example, olive oil is nutrient-dense and abundant in unsaturated fats such that 14 g of olive oil contains 119 calories of which 73% are monounsaturated fat and 11% are polyunsaturated fat [112].

### 8.7. Module 9—Essential Fatty Acids

While non-essential fatty acids can be produced through a series of biochemical pathways, omega 3 and 6 (n-3 and n-6, respectively) essential polyunsaturated fatty acids cannot be synthesized in the body and must be obtained through the diet [113]. There are two main types of n-3 fatty acids, those found in fish oils (eicosapentaenoic acid and docosahexaenoic acid) and those derived from plant oils (alpha-linolenic acid) [114]. Table 1 presents the recommended intakes, functions, and examples of n-3 and n-6 fatty acids.

#### 8.7.1. N-3 and N-6 Essential Fatty Acid Intakes

Among persons with SCI, the evidence in the literature indicates poor intake of n-3 and n-6 fatty acids and suggests sex as a determinant for intake of n-3 fatty acids. Iyer et al. [90] reported that intake of n-3 alpha-linolenic acid was lower in males than in females with SCI, with an overall compliance of 35% of adequate intake recommendations. The same authors [90] also reported low n-6 linoleic acid intake among persons with SCI, with 90% not meeting adequate intake recommendations. Similarly, Groah et al. [67] reported that women with SCI exceeded or approached the recommended intake of n-3 linolenic acid of 1.1 g/d, whereas all men with SCI in the study had lower than the recommended intake of 1.6/d with an average intake of half the recommendations (0.8 g/d). In the same study, the authors [67] also reported that 73 community-dwelling persons with SCI had a mean intake of n-6 linoleic acid lower than the recommendations of 17 and 11 g/d for men and women, respectively.

#### 8.7.2. Health Benefits of N-3 Essential Fatty Acids

N-3 fatty acids have been shown to reduce inflammation, prevent cardiovascular disease, support brain function, maintain healthy cholesterol profiles, support the immune system, and reduce obesity [113,115,116]. Insufficient levels of essential fatty acids and an imbalance of the n-3/n-6 fatty acid ratio are associated with dyslipidemia, high blood pressure, heart disease, and an increased risk for premature death in the population without SCI [117]. Among persons with SCI, n-3 fatty acids have been gaining attention due to the role they play in the induction of neuro recovery effects related to their anti-inflammatory and antioxidant properties [118]. Moreover, n-3 fatty acids have been demonstrated to have neuroprotective and neurogenerative effects in most animal models of SCI [119,120]. The translation of the positive results of n-3 fatty acid from animal models to studies in humans with SCI is currently underway [118].

In an RCT, Allison et al. [121] assessed the effect of a three-month anti-inflammatory diet in 20 persons with SCI compared to a control group. The authors [121] reported that an increase in the intake of n-3 fatty acids was associated with a reduction of proinflammatory mediators (interferon-y, interleukin-1β, and interleukin-6) in the treatment group. Furthermore, this group [122] also demonstrated an increased consumption of n-3 fatty acids in persons with SCI after three months of following a diet consistent with the healthy dietary patterns presented in the DGA. In contrast, Sabour et al. [123] examined the effects of n-3 fatty acids on inflammatory cytokines in 75 osteoporotic persons with SCI. The authors [123] did not observe any influence of n-3 docosahexaenoic and eicosapentaenoic acids on inflammatory markers after 4 months of supplementation. In another report, Sabour et al. [124] evaluated the effect of n-3 docosahexaenoic and eicosapentaenoic acids on circulatory concentrations of leptin and adiponectin (adipose-derived biomarkers related to cardiovascular disease risk) in 104 persons with SCI. The authors [124] did not observe any significant influence on leptin levels after 14 months; however, adiponectin levels were significantly reduced.

#### 8.7.3. N-6 Fatty Acid and its Influence on Cardiovascular Disease Risk

There is evidence that support the importance of n-6 fatty acids for optimal health, however, excess intake may be harmful. N-6 fatty acids are involved in the regulation of energy production as part of metabolism, as well as the maintenance of skin, bone, and hair health [125]. Although n-6 fatty acids are vital for several physiological functions, their effect on cardiovascular health remains unclear [116,125]. Some evidence indicates that an increased intake of n-6 fatty acids can lower cardiovascular disease risk by reducing total serum cholesterol, lowering blood pressure, improving insulin resistance, and reducing incidence of diabetes mellitus [125,126,127]. However, it is unclear if the cardio-protective benefits observed are related to the increased intake of n-6 fatty acids or the replacement of saturated fats [127]. Nevertheless, the cholesterol-lowering effect of n-6 fatty acids is well-established; the increased intake of n-6 fatty acids was demonstrated to be greater than the effect produced by the removal of saturated fatty acids alone [128].

On another note, there are concerns that high intakes of n-6 fatty acids may increase cardiovascular disease risk by increasing inflammation [125]. Intake of linoleic acid, the predominant n-6 fatty acid in the Western diet, has recently increased substantially primarily due to recommendations claiming the health benefits of lowering cholesterol levels with increased consumption linoleic acid [126]. However, linoleic acid elongates and desaturates to form arachidonic acid, a precursor to pro-inflammatory proteins [126]. High intakes of linoleic acid can increase the susceptibility of low-density lipoprotein to oxidation which then promotes vascular inflammation [127]. 

To offset this potential side-effect of increased linoleic acid consumption, a widely accepted approach is to counteract the proinflammatory effects with n-3 fatty acids [126]. A high n-6/n-3 ratio promotes the pathogenesis of several diseases, including cardiovascular disease, cancer, inflammatory, and autoimmune diseases, whereas increased intakes of n-3 fatty acids (i.e., a lower ratio) have suppressive effects [129]. The Western diet is comprised of a n-6/n-3 ratio of 15/1–16.7/1; however, n-6/n-3 ratios ranging from 2/1–5/1 have been reported to have beneficial effects of reduced rectal cell proliferation in patients with colorectal cancer, decreased breast cancer risk, suppressed inflammation in patients with rheumatoid arthritis, and a beneficial effect on patients with asthma [129]. Regarding cardiovascular disease risk, a ratio of 4/1 was associated with a 70% decrease in total mortality [129].

### 8.8. Module 10—Ways to Eat Less Fat and Fewer Calories

The reduction of unhealthy fat intake is an effective mechanism of reducing overall energy intake and improving diet quality. However, education on how to reduce fat intake and improve diet quality is necessary for effective behavioral change for both persons with and without SCI. Kressler et al. [130] noted that the use of behavioral strategies to facilitate adherence is needed as part of effective lifestyle interventions to reduce cardiometabolic disease in persons with SCI. Therefore, tools and techniques must be provided alongside education to assist with effective behavioral change. Following are three suggested ways to reduce fat and energy intake, which will yield substantial cumulative results especially if all three methods are implemented and maintained long-term.

#### 8.8.1. Eat High-Fat, Energy-Dense Foods Less Often

The first method of reducing fat intake is to eat high-fat, energy-dense foods less often. For example, if a person typically consumes a large serving of French fries (which contains 23 g of fat) each day, it would be recommended that they only indulge in the food item once a week, resulting in a reduction of 138 g of fat per week (23 g × 6 days). This practice alone will reduce energy intake by 1242 calories per week (23 g fat × 9 calories/g) or 64,584 calories per year (1242 calories per week × 52 weeks in a year). With one pound of fat being produced with an excess of 3500 calories [131], the reduction of close to 65,000 calories per year equates to the prevention of ~18 lbs. of lipogenesis.

This recommendation allows an individual to continue to enjoy certain calorie-dense foods, but with the understanding that they will eat them sparingly. Dietary guidelines by Farkas et al. [2] support this recommendation for persons with SCI, stating that high-fat, energy-dense foods should be consumed on special occasions, and not be a regular occurrence.

#### 8.8.2. Eat Smaller Portions of High-Fat, Energy-Dense Foods

The second method of reducing fat intake involves restricting the portion sizes of high-fat, energy-dense foods. For example, rather than ordering a large serving of French fries containing 23 g of fat, ordering a medium size with 15 g of fat would result in a reduction of 8 g of fat or 72 kcals. This method can eventually be progressed by reducing the order to a small serving of French fries, thereby furthering energy restriction.

#### 8.8.3. Substitute with Lower-Fat, Lower-Energy Foods

Lastly, replacing high-fat, energy-dense foods with lower-fat, lower-calorie food items will further reduce energy intake. Simple substitutions like replacing French fries with baked fries will reduce the energy content by ~60 kcals per cup. Fruits and vegetables are ideal replacements considering their low-energy, nutrient-dense profile; however, other effective substitutions include exchanging full-fat milk with reduced-fat milk, regular salad dressing with reduced-fat dressing, full-fat yogurt and cheese with reduced-fat yogurt and cheese, and potato chips with pretzels or dehydrated fruit. Other methods of reducing fat content include trimming off the fat on meats, removing the skin on poultry, and using preparation methods requiring minimal added fat such as grilling, braising, baking, and roasting compared to deep frying. To quantify the fat reduction utilizing this method, a serving of fried chicken contains 21 g of fat compared to a serving of grilled chicken containing 7 g of fat. This equates to a reduction of 14 g of fat or 126 kcals per serving.

### 8.9. Module 11—Carbohydrates

Carbohydrates provide the body’s primary source of energy and the brain’s preferred energy source. All carbohydrates (simple and complex) are broken down into glucose which is metabolized to fuel the body’s cells, tissues, and organs. The AMDR for carbohydrates is 45–65% of total daily calories [14]. Table 1 presents the different forms of carbohydrates, recommended intakes, and examples of sources.

#### 8.9.1. Current Carbohydrate Intake

Among persons with SCI, several studies demonstrate that approximately half of the total daily energy consumed were sourced from carbohydrates. Nightingale et al. [132] and Silveira et al. [50] reported 44% and 49% of total energy intake, respectively, were sourced from carbohydrates in individuals with SCI. Sabour et al. [133] reported that more than half the calories (53%) were sourced from carbohydrates in 162 persons with SCI, mainly from simple carbohydrates. Walters et al. [134] reported carbohydrate intake of 52% for men and 53% for women with SCI. Similarly, Levine et al. [21] demonstrated carbohydrate intake of 46% for men and 51% for women with SCI. Farkas et al. [29] in a recent meta-analysis among populations with SCI, reported a pooled percentage of 52% for total carbohydrate intake which was within the 2015–2020 DGA recommendations.

Although total carbohydrate intake may be meeting recommendations, the intake of nutrient-dense carbohydrate food sources, including whole grains, fruits, and vegetables is reported to be low in both persons with and without SCI [18,50]. Silveira et al. [50] reported that persons with SCI consumed significantly fewer whole-grains than persons without SCI. Lieberman et al. [54] demonstrated similar findings, where the daily servings of whole-grains were significantly lower in persons with SCI compared to controls without SCI. They also showed that 9% of individuals with SCI met DGA’s recommendations for whole-grain intake of at least 3 ounces per day compared to 21% of age-sex matched controls [54]. Carbohydrates should be sourced primarily from complex carbohydrates which are rich in micronutrients and fiber that help to control blood sugar [135], limit rapid spikes in blood glucose and insulin [135,136], aid with satiation/satiety [2] and facilitate bowel programs by adding bulk to stools [2].

#### 8.9.2. Health Benefits of Complex Carbohydrates

Population studies in people without SCI have demonstrated the positive association of whole-grain consumption and lowered risk of heart disease, cancer (especially colon), and inflammatory bowel disease [137]. Dietary fiber from complex carbohydrates slow the absorption rate of glucose contributing to the lowering of the postprandial glucose peak [138]. This is especially important for persons with prediabetes or diabetes who have difficulty with hyperglycemia and controlling blood sugar levels. Among persons with SCI, Goldsmith et al. [139] recently reported a negative association of carbohydrate intake with several measures of fat mass, where higher carbohydrate intake was associated with lower fat mass in 48 individuals with SCI.

### 8.10. Module 12—Fiber

Dietary fiber is nondigestible carbohydrates and lignin found in plant-based food only [14,140]. Benefits of fiber include maintaining satiety/satiation, managing weight, aid with digestion, and decreasing the risk for certain cancers [141]. Fiber yields an average of 1.5–2.5 calories per gram; however, providing energy for the body to use is not a significant function of fiber [2]. Fiber is subdivided into soluble fiber and insoluble fiber. Soluble fiber helps lower cholesterol and control blood glucose while insoluble fiber adds bulk to stool by absorbing water to assist moving the stool through the gastrointestinal tract [140,141]. Aune et al. [65] in a meta-analysis demonstrated the inverse association of whole-grain (fiber-containing) consumption and risk of coronary heart disease, cardiovascular disease, total cancer, and mortality from all causes, respiratory diseases, infectious diseases, diabetes, and all non-cardiovascular, non-cancer causes.

#### 8.10.1. Fiber Recommendations

2020–2025 DGA recommendations for dietary fiber intake is 14 g/1000 kcals consumed; thus 28 g are needed in a 2000 kcal/day diet [18]. There are no specific DGA recommendations for fiber intake in persons with SCI. Consuming just the right amount of fiber is challenging for individuals with and without SCI as insufficient fiber intake may lead to constipation or bowel impaction, while excess fiber intake can lead to painful bloating and excess gas [18,141]. The general recommendation by the AND is to gradually increase fiber intake over a period to allow for the gastrointestinal system to adjust.

The AND’s [30] recommendations for increasing fiber intake in individuals with SCI are to start with about 15 g per day and slowly increase intake to 30 g per day if tolerated. If intolerance symptoms occur (i.e., abdominal pain and/or constipation), a reduction of fiber intake is recommended [30]. To prevent constipation with increased fiber intake, commensurate water should be consumed [30].

#### 8.10.2. Current Fiber Intake

Considering the evident shortfall in consumption of whole fruits, vegetables, and whole-grain intake, all of which are important contributors to dietary fiber, it is not surprising that more than 90% of individuals without SCI do not meet the recommended dietary fiber intake [18,140]. Similarly, individuals with SCI are reported to have inadequate intake of complex carbohydrates [50,54], contributing to inadequate dietary fiber intake according to recommendations for the population without SCI [21,29,49,67,133]. Levine et al. [21] found dietary fiber intake among persons with SCI to be 25% of recommended levels. Silveira et al. [50] found fiber intake in persons with SCI to marginally meet the AND’s minimum recommendation of 15 g/day. Similarly, Farkas et al. [29] found fiber intake in persons with SCI inadequate. Iyer et al. [90] is the only study that reported a high fiber consumption ranging from 30-33g/day while other studies report dietary intakes ranging from 12–22 g/day in persons with SCI [21,29,49].

#### 8.10.3. Fiber and Neurogenic Bowel

Lower fiber intake among persons with SCI compared to persons without SCI is likely due to SCI-specific factors such as fiber’s potential adverse effects on the bowel [29,64]. Neurogenic bowel dysfunction (NBD) refers to the dysregulation of the bowel as a result of loss of central nervous system input [64]. Lower gastrointestinal dysfunction, such as constipation and fecal incontinence, is common in persons with chronic SCI, with reported prevalence between 75–80% [64,142]. The validated NBD score is a condition-specific tool to assess the quality of life impacted by NBD in persons with SCI [143]. Fiber intake has been reported to affect NBD score [144]. The amount and type of food has been reported to change following SCI in the context of NBD [145]. Increased cases of NBD have been reported due to high fiber intake, which may explain the tendency to consume foods deficient in fiber [64].

Cameron et al. [146] reported that increasing the dietary fiber in individuals with SCI does not have the same effect on bowel function as those who are neurologically intact with a normally functioning bowel. In fact, the effect of a high-fiber diet in persons with SCI may be contrary to the desired effect. Moreover, to prevent constipation associated with increased fiber intake, a proportionately higher intake of water should be consumed; this further complicates the situation for individuals with SCI who will have to cath more often to prevent leakage and/or accidents [2].

#### 8.10.4. Proposed Fiber Requirements in SCI

The average energy intake reported for persons with SCI ranges from 1212 to 2732 kcals/day [26,36,50,67,132,133,147,148,149,150]. In a recent meta-analysis, Farkas et al. [29] reported a pooled energy intake of 1876 kcal/day. Incorporating the DGA’s fiber recommendations of 14 g/1000 kcals, this would amount to the recommended intake of 26 g/d when using the pooled energy intake, which is lower than the recommended amount of 28–34 g/day for the average adult (sex and age-dependent) [30]. Rather than using the amount of energy consumed to determine fiber requirements, calculating energy needs as a basis for determining fiber requirements in persons with SCI may be more suitable considering that they may be in energy imbalance, and overestimating fiber requirements may increase complications for neurogenic bowel. Therefore, the accurate determination of TDEE via indirect calorimetry and the 1.15 correction factor [36] is crucial to accurately determine fiber requirements. If indirect calorimetry is unavailable, SCI-specific energy expenditure equations may be used [34,35].

### 8.11. Module 13—Planning a Healthy Breakfast

Breakfast is the first meal consumed within 2 h of waking up and is commonly regarded as the most important meal of the day. Breakfast contributes to 20–35% of the total daily energy requirement [151]. While some believe skipping breakfast may help with fat reduction, evidence demonstrates otherwise [151,152,153]. Among persons without SCI, Ma et al. [151] demonstrated a significant association between skipping breakfast and obesity in a systematic review and meta-analyses. In another meta-analysis, Takagi et al. [153] observed that skipping breakfast is significantly associated with an increased risk for heart disease [153]. Bonnet et al. [152] demonstrated that skipping breakfast was significantly associated with increased low-density lipoprotein cholesterol and cardiovascular disease risk. To date, no studies have investigated the consumption of breakfast meals and their association with obesity and metabolic health outcomes in persons with SCI.

The skill of putting together a healthy breakfast meal should be fostered in individuals with SCI, with MyPlate serving as a guide for a nutritious and balanced meal. An emphasis is placed on incorporating vegetables into breakfast meals, which are mainly consumed at lunch and dinner. Easy ways to include vegetables in breakfast meals are to add them to egg dishes (e.g., omelets, scrambled eggs, etc.), breakfast sandwiches and burritos, or blend them in a smoothie. This module also highlights the need to limit energy-dense yet nutrient-poor items; common breakfast items that should be avoided are muffins, doughnuts, sugar-loaded cereal, butter, syrup, cream cheese, pastries, coffeecake, and sugar-rich coffee drinks.

### 8.12. Module 14—Building a “Light” Meal

Excess energy intake is a driving factor for neurogenic obesity among individuals with SCI [2,25]. Therefore, a vital skill to foster is the manipulation of a meal’s energy density while still making it balanced per the five MyPlate Groups. For example, if a person with SCI eats a large meal for lunch, they can create a smaller, lighter meal for dinner that is nutrient-dense but energy-light. Such light meals contain more calories than snacks but not as much as full-fledged meals. Creating light meals aims to stay within energy limits and prevent excessive intake. Based on USDA’s MyPlate recommendations, an example of a light meal is a whole-grain sandwich with low-energy protein (i.e., water packed tuna fish, chicken breast, etc.), five baby carrots, half a cup of fruit, and one cup of low-fat milk.

### 8.13. Module 15—Satisfying Snacks

While there is no consistent definition for a snack [154], the consensus is that a snack is anything consumed outside the traditional meals of breakfast, lunch, and dinner [155]. However, it is inconclusive if those extra eating occasions are considered meals or snacks [155]. Contributing to approximately a third of total daily calories, snacks are currently being investigated as a factor in the obesity pandemic for persons without SCI [156,157,158], and should also be considered in those with SCI. Sebastian et al. [157] reported that the average number of snacks consumed per day has doubled over the past several decades, the percentage of adults without SCI that eat snacks nearly doubled, and that snacking more times throughout the day was associated with increased energy intake [157]. Berteus- Forslund et al. [158] observed that obese subjects snacked significantly more often than the healthy weight reference group and that energy intake increased with increased snack frequency, irrespective of physical activity. The authors from both studies [157,158] note that chosen snacks were typically energy-dense yet nutrient-poor (e.g., sweet, fatty food) and contributed considerably to energy intake.

Recommendations for healthy snacks are those that are low in calories (~200 calories,) and nutrient dense. The best snack options are fruits and vegetables, given that they are naturally energy-light, nutrient-dense, and easily accessible with little to no preparation. Other healthy options include air-popped popcorn without butter, lightly salted pretzels, rice cakes, 100-calorie packs of unsalted nuts/trail mix, dried fruit with no added sugar, whole-grain toast, low-fat cottage cheese, yogurt, and frozen yogurt. This is especially practical for persons with SCI who could benefit from easily accessible and prepared, healthy snacking practices. While there is ample research on the practice of snacking and its role in driving obesity in persons without SCI, there are no studies investigating this in individuals with SCI. Nevertheless, education on healthy snacking should be routinely provided to persons with SCI who would benefit from easily accessible, nutrient-dense foods to improve overall dietary patterns and lower total energy intake.

### 8.14. Module 16—Reading the Nutrition Facts Label

The nutrition facts label is a valuable tool for making better dietary choices by providing ample information and a list of ingredients [159]. However, the nutrition facts label is under-utilized despite its intended purpose [160]. Studies have shown that most consumers without SCI do not know how to read and understand nutrition facts labels [160,161,162,163,164]. Blitstein and Evans [164] found that only 53% of consumers without SCI reported using nutrition facts labels. The same authors [164] also found that females, those with higher education, and those currently married were more likely to use the nutrition facts label. Graham et al. [161] demonstrated that the front-of-package nutrition label was more likely to be read than the nutrition facts label on the side or back of the package. The authors [161] concluded that education should be provided to increase the utilization of the nutrition facts label. Low reported usage is likely due to the daunting nutrition information provided in the context of minimal education for use.

Among persons with SCI, Wood et al. [165] evaluated the effect of a tele-nutrition counseling program on diet quality, weight, waist circumference, and quality of life in 15 persons with SCI. The authors [165] reported significant improvement in reading the nutrition facts label after the three-month intervention. Limited research is available on the utilization of the nutrition facts labels in persons with SCI; however, there are authors [130,166] who support its use as a behavioral modification for lifestyle intervention in reducing cardiometabolic disease in SCI. Kressler et al. [130] noted that the best way to measure energy intake is to extract those values from food labels; however, this method is tedious and time-consuming, especially for persons with limited hand function.

To improve readability, the nutrition fact label was redesigned in 2016 [159]. Changes to the nutrition facts included bolded serving sizes, enlarged print for calories per serving, and including a new section for added sugars, as well as a new footnote explaining the daily calorie intake upon which the nutrition percentages are based (i.e., 2000 kcal/day) [159]. [159] Despite these changes, Gibbs et al. [162] found the redesign was not easier to read among consumers in the general population with similar nutrition literacy. These findings emphasize the necessity for education to increase nutrition label utilization. Basic skills like arithmetic and knowledge of nutrition components are necessary since nutrition facts labels naturally contain abundant numbers requiring calculations. Persoskie et al. [163] demonstrated the need for education, including mathematics, for nutrition label understanding. The same authors [163] also noted the correlation between label understanding and self-reported dietary behaviors, with higher scores for label understanding being significantly associated with consuming more vegetables and less sugar-sweetened soda. A positive association was also observed between fruit intake and higher scores of nutrition label understanding, although not significant [163]. Miller et al. [160] demonstrated that nutrition knowledge supports food label use, which is consistent with the cognitive processing model that asserts that individuals with prior knowledge and understanding are more likely to use the label information effectively. The same authors [160] noted that consumers with previous knowledge could focus on salient information, understand information, and make healthful decisions based on the information presented on the labels.

## 9. Conclusions

A myriad of SCI-specific nutritional adjustments must be considered when providing dietetic care to individuals with SCI. Because persons with SCI experience markedly reduced TDEE and energy needs, we emphasize the importance of measuring or estimating energy expenditure with SCI-specific prediction equations to determine energy and nutritional needs. The cumulative effects of overfeeding exacerbate SCI-associated comorbidities and overconsumption of foodstuff if energy balance is not immediately attained and sustained. To account for the substantial reduction in TDEE, a low-energy, nutrient-dense dietary pattern according to the DGA is recommended for persons with SCI because of its cardioprotective properties. While this review focused on nutrition, the authors also emphasize the health benefits associated with physical exercise that when coupled with a healthy dietary pattern provides a stronger benefit.

Current evidence indicates that the diet quality of persons with SCI is similar to or worse than that of the population without SCI, and consequently improvements are needed. Ideally, persons with SCI are referred to a registered dietitian for nutritional counseling and therapy; however, it is not always feasible because not all registered dietitians have expertise in SCI medicine to provide adequate nutritional care. We therefore proposed that the nutrition education modules detailed in this manuscript be provided to health care professionals (including dietitians in direct care of persons with SCI) and consumers to mitigate several SCI-related comorbidities and improve dietary knowledge and profiles.

Each nutrition module presented here within provides a comprehensive nutrition education guide. The first module introduces the MyPlate consumer guide based on the DGA recommendations as an excellent tool to present the five food groups as part of a healthy dietary pattern. Modules two through seven provide further detail of each food group with key recommendations, including half the plate comprising of fruits and vegetables, approximately a quarter of the plate containing grains (e.g., rice, bread, pasta), a quarter of the plate with lean protein (e.g., eggs, chicken breast, turkey), and a cup of low-fat dairy (e.g., milk, cheese, yogurt). In modules eight, nine, and 11, fats and carbohydrates are explained and emphasize the importance of essential fatty acids and whole-grain consumption. Module 12 discusses fiber recommendations, identifying whole-grain as a good source of fiber, and fiber’s importance with regard to neurogenic bowel. Lastly, modules 10 and 13–16 offer techniques that facilitate the implementation and maintenance of the module recommendations.

While these modules serve as a foundation to increase nutrition literacy, they can be further evolved. For example, the use of herbs and spices can be used to lower sodium intake and add flavor in place of fatty additives. These modules should be incorporated into a global, comprehensive medical approach factoring in an individual’s body composition, comorbidities, current dietary intake, and energy expenditure to achieve optimal health. Future research should aim to test the nutrition education modules in persons with SCI with a specific focus on increasing SCI-specific nutrition and health knowledge in persons with SCI, their caregivers, and healthcare providers, as well as reducing neurogenic obesity and its associated comorbidities.

## Figures and Tables

**Table 1:** Macronutrient and Water Nutrition Education Reference for Health Care Professionals and Consumers.

Nutrient	Recommendations	Function	Sources
Macronutrients (energy content)			
Carbohydrates (4 kcal/g)	45–65% ^∀,Δ^	Primary source of energy for cells; brain’s preferred fuel source [14].	
Simple Carbohydrates	Limit		Refined sugars, candy, syrups, soft drinks, etc.
Monosaccharide			
Glucose			
Fructose			
Galactose			
Disaccharide			
Sucrose			
Lactose			
Maltose			
Complex Carbohydrates	At least 50% of total grain intake should be whole grain.^Δ^		Grains (whole, refined), vegetables, beans, peas, etc.
Polysaccharide			
Starch			
Cellulose			
Protein (4 kcal/g)	10–35% ^∀,Δ^	Supplies amino acids which are the major structural components of cells. Needed to serve as enzymes, hormones, membrane receptors, and transporters of nutrients [14].	Meats, poultry, fish, eggs, dairy, beans, etc.
Essential amino acids			
Histidine	≥19 y: 14 mg/kg/d ^	Helps with histamine production, immunity, digestion, sleep, and sexual function [15].	Meat, fish, poultry, nuts, seeds, whole grains, etc.
Isoleucine	≥19 y: 19 mg/kg/d ^	Involved in muscle metabolism, immune function, produces hemoglobin, and regulates energy [15].	Meat, fish, poultry, eggs, cheese, lentils, nuts, seeds, etc.
Leucine	≥19 y: 42 mg/kg/d ^	Essential for protein synthesis and growth hormones, grows and repairs muscle tissue, heals wounds and regulates blood sugar [15].	Dairy, soy, beans, legumes, etc.
Lysine	≥19 y: 38 mg/kg/d ^	Hormone production, cell division, fat metabolism, and wound healing [15].	Meat, eggs, soy, black beans, quinoa, pumpkin seeds, etc.
Methionine	≥19 y: 19 mg/kg/d ^	Metabolism, detoxification, tissue growth, absorption of zinc and calcium [15].	Eggs, grains, nuts, seeds, etc.
Phenylalanine	≥19 y: 33 mg/kg/d ^	Production of dopamine, epinephrine, norepinephrine, and other amino acids [15].	Dairy, meat, poultry, soy, fish, beans, nuts, etc.
Threonine	≥19 y: 20 mg/kg/d^	Needed for collagen and elastin synthesis, clot formation, fat metabolism, and immune function [15].	Cottage cheese, wheat germ, etc.
Tryptophan	≥19 y: 5 mg/kg/d^	Nitrogen balance, serotonin production [15].	Wheat germ, cottage cheese, chicken, turkey, etc.
Valine	≥19 y: 24 mg/kg/d^	Muscle growth, tissue regeneration, and providing energy [15].	Soy, cheese, peanuts, mushrooms, whole grains, vegetables, etc.
Non-essential amino acids			
Alanine	N/A	Nervous system function, tryptophan synthesis [16].	Meat, poultry, fish, eggs, dairy, etc.
Asparagine	N/A	Glycoprotein synthesis, liver health; central nervous system signaling and development; Energy levels [16].	Dairy, beef, poultry, eggs, fish, seafood, potatoes, legumes, nuts, seeds, soy, whole grains, etc.
Aspartic Acid	N/A	Precursor to other amino acids in the citric acid and urea cycles; serves as an excitatory spinal cord neurotransmitter [16].	Oysters, sprouting seeds, oat flakes, avocado, asparagus, etc.
Glutamic Acid	N/A	Precursor to gamma-aminobutyric acid; Excitatory neurotransmitter [16].	Meat, poultry, fish, eggs, dairy, high-protein vegetables, etc.
Conditionally essential amino acids			
Arginine	N/A	Increases T-cell production; release of insulin and human growth hormones, neutralize hepatic ammonia, and ameliorate skin/connective tissue quality and healing [16].	Meat, fish, nuts, seeds, legumes, whole grains, dairy, etc.
Cysteine	N/A	Protein and coenzyme A synthesis, glutathione production [16].	Chickpeas, couscous, eggs, lentils, oats, turkey, walnuts, etc.
Glutamine	N/A	Nervous system, increase energy supply [16].	Chicken, fish, cabbage, spinach, dairy, tofu, lentils, beans, etc.
Tyrosine	N/A	Dopamine and noradrenaline precursor; protein synthesis [16].	Soy, chicken, turkey, fish, peanuts, almonds, avocados, bananas, milk, cheese, yogurt, cottage cheese, lima beans, pumpkin seeds, sesame seeds, etc.
Glycine	N/A	Collagen, sleep, central nervous system [16].	Red meat, seeds, turkey, chicken, pork, peanuts, granola, etc.
Proline	N/A	Collagen synthesis; provides energy through collagen degradation under stress [16].	Bone broth, chicken wings (with skin), pork rinds, gelatin, etc.
Serine	N/A	Neuromodulatory role; creatine, epinephrine, DNA, and RNA production [16].	Soybeans, nuts, eggs, chickpeas, lentils, meat, fish, etc.
Fats (9 kcal/g)	20–35% ^∀,Δ^	Provides energy, supports cell function, absorption of fat-soluble nutrients, hormone synthesis [14].	
Unsaturated Fat	27 g of oil ^Δ^; Replace saturated fat with unsaturated fat. ^Δ,α^		
Monounsaturated fat			Avocado, nuts, seeds, oils (e.g., olive oil), etc.
Polyunsaturated fat			
Linolenic acid (Omega-3 essential fatty acid)	Females: ≥19 y: 1.1 g ^ Males: ≥19 y: 1.6 g ^; 0.6–1.2% ^∀^	Eicosapentaenoic acid and docosahexanoic acid and n-3 eicosanoids precursor [14]	Salmon, mackerel, chia, flax, walnuts, etc.
Linoleic acid (Omega-6 essential fatty acid)	Females: 19–50 y: 12 g ^^,Δ^ ≥51 y: 11 g ^^,Δ^ Males: 19–50 y: 17 g ^^,Δ^ ≥51 y: 14 g ^^,Δ^; 5–10% ^∀^	Arachidonic acid precursor, membrane structural lipid component, cell-signaling pathways [14].	Corn oil, soybean oil, safflower oil sunflower oil, nuts, etc.
Saturated Fat	<10% ^Δ^; 5–6% ^α,ϑ^		Milk, cheese, red meat, butter, coconut oil, etc.
Trans-Fat	Minimal as possible		
Natural trans-fat	Limit		Animal products
Artificial trans-fat	Avoid		Fully or partially hydrogenated oil
Alcohol (7 kcal/g) [17]	Do not start drinking, limit, or drink in moderation (Females: ≤1 drink/d; Males: ≤2 drinks/d)	Not an essential macronutrient; adults of legal drinking age can choose to not drink alcohol [18].	Beer, wine, liquor
Water (0 kcal/g)	Females: ≥19 y: 9 cups/d ^η^ Males: ≥19 y: 13 cups/d ^η^	Carries nutrients and oxygen to cells; lubricates joints; lessens burden on kidneys and liver by removing waste products; helps dissolve minerals and nutrients to make them accessible	Soup, milk, tea, coffee, juice, and drinking water

^∀^ Acceptable Macronutrient Distribution Range; ^Δ^ 2020–2025 United States Department of Agriculture Dietary Guidelines for Americans; ^ Recommended Daily Allowance; ^α^ American Heart Association; ^ϑ^ Paralyzed Veterans of America Clinical Practice Guidelines on the Identification and Management of Cardiometabolic Risk After Spinal Cord Injury; ^η^ Institute of Medicine/National Academy of Medicine.

**Table 2:** Micronutrients (Vitamins and Minerals) Nutrition Education Reference for Health Care Professionals and Consumers.

Nutrient	Recommendations	Function	Sources
Vitamins			
Water-soluble vitamins			
Vitamin B1 (Thiamin)	Females: ≥19 y: 1.1 mg ^Δ,^^ Males: ≥19 y: 1.2 mg ^Δ,^^	Helps cells convert carbohydrates into energy [19].	Watermelon, acorn squash, etc.
Vitamin B2 (Riboflavin)	Females: ≥19 y: 1.1 mg ^Δ,^^ Males: ≥19 y: 1.3 mg ^Δ,^^	Works with other B vitamins, red blood cell growth and production [19]	Milk, yogurt, cheese, whole and enriched grains, etc.
Vitamin B3 (Niacin)	Females: ≥19 y: 14 mg ^Δ,^^ Males: ≥19 y: 16 mg ^Δ,^^	Maintain healthy skin and nerves [19].	Meat, poultry, fish, fortified/whole grains, mushrooms, potatoes, etc.
Vitamin B5 (Pantothenic acid)	≥19 y: 5 mg ^	Food metabolism, hormone and cholesterol production [19].	Chicken, whole grains, broccoli, avocados, mushrooms, etc.
Vitamin B6 (Pyridoxine)	Females: 19–50 y: 1.3 mg ^Δ,^^; ≥ 51 y: 1.5 mg ^Δ,^^ Males: 19–50 y: 1.3 mg ^Δ,^^; ≥51 y: 1.7 mg ^Δ,^^	Red blood cell formation, maintains brain function [19].	Meat, fish, poultry, legumes, soy, bananas, etc.
Vitamin B7 (Biotin)	≥19 y: 30 mcg ^Γ^	Protein and carbohydrate metabolism, hormone and cholesterol production [19].	Whole grains, eggs, soybeans, fish, etc.
Vitamin B9 (Folate/folic acid)	≥19 y: 400 mg ^Δ,^^	Forms red blood cells with vitamin B12; DNA synthesis [19].	Fortified grains and cereals, asparagus, spinach, broccoli, black-eyed peas, chickpeas, etc.
Vitamin B12 (Cobalamin)	≥19 y: 2.4 mcg ^Δ,^^	Involved with metabolism, red blood cell formation, maintenance of central nervous system [19].	Meat, poultry, fish, milk, cheese, fortified soymilk, and cereals, etc.
Vitamin C (Ascorbic acid)	Females: ≥19 y: 75 mg ^Δ,^^ Males: ≥19 y: 90 mg ^Δ,^^	Antioxidant, healthy teeth and gums, iron absorption, maintains healthy tissue, wound healing [19].	Citrus fruits, potatoes, broccoli, bell peppers, spinach, strawberries, tomatoes, Brussel sprouts, etc.
Fat-soluble vitamins			
Vitamin A	Females:≥19 y: 700 mcg RAE ^Δ,^^ Males: ≥19 y: 900 mcg RAE ^Δ,^^	Forms/maintains healthy teeth, bones, soft tissue, mucous membranes, and skin [19].	Beef, liver, eggs, shrimp, fish, fortified milk, sweet potatoes, carrots, pumpkin, spinach, mangoes, etc.
Vitamin D	Females and Males: 19–70 y: 600 IU ≥71 y: 800 IU ^Δ,^^	Calcium absorption, maintains blood calcium and phosphorus levels [19].	Fortified milk and cereals, fatty fish, etc.
Vitamin E	Females and Males: ≥19 y: 15 mg AT ^Δ,^^	Antioxidant, red blood cells formation, vitamin K usage [19].	Vegetable oils, leafy green vegetables, whole grains, nuts, etc.
Vitamin K	Females: ≥ 19 y: 90 mcg ^Δ,Γ^ Males: ≥19 y: 120 mcg ^Δ,Γ^	Assists with blood coagulation [19].	Cabbage, eggs, milk, spinach, broccoli, kale, etc.
Minerals			
Macrominerals (Major)			
Calcium	Females: 19–50 y: 1000 mg ^Δ,^^; ≥51 y: 1200 mg ^Δ,^^ Males: 19–71 y: 1000 mg ^Δ,^^; ≥71 y: 1200 mg ^Δ,^^	Bone and teeth health, muscle and nerve function, blood clotting, blood pressure regulation, immune system health [20].	Yogurt, cheese, milk, salmon, leafy green vegetables, etc.
Chloride	Females and Males: 19–50 y: 2300 mg ^ 50–70 y: 2000 mg ^ ≥70 y: 1800 mg ^	Fluid balance, stomach acid [20].	Salt
Magnesium	Females: 19–30 y: 310 mg ^Δ,^^; ≥31 y: 320 mg ^Δ,^^ Males: 19–30 y: 400 mg ^Δ,^^; ≥31 y: 420 mg ^Δ,^^	Bone health, protein metabolism, muscle contraction, nerve transmission, immune system health [20].	Spinach, broccoli, legumes, seeds, whole-wheat bread, etc.
Potassium	Females: ≥19 y: 2300 mg ^Δ,Γ^ Males: ≥3400 mg ^Δ,Γ^	Fluid balance, nerve transmission, and muscle contraction [20].	Meat, milk, fruits, vegetables, grains, legumes, etc.
Sodium	≥19 y: ≤2300 mg ^Δ,δ^ Persons with SCI and HTN: ≤2400 mg ^ϑ^ Ideal: ≤1500 mg ^α^	Fluid balance, nerve transmission, and muscle contraction [20].	Salt, soy sauce, vegetables, canned food, etc.
Phosphorus	Females and Males: ≥19 y: 700 mg ^Δ,^^	Bone and teeth health; acid-base balance [20].	Meat, fish, poultry, eggs, milk, etc.
Microminerals (Trace)			
Chromium	Females: 19–50 y: 25 mcg ^Γ^; ≥51 y: 20 mcg ^Γ^ Males: 19–50 y: 35 mcg ^Γ^; ≥51y: 30 mcg ^Γ^	Works with insulin to regulate blood glucose [20].	Meat, poultry, fish, nuts, cheese, etc.
Copper	≥19 y: 900 mcg ^	Enzymes, iron metabolism [20].	Shellfish, nuts, seeds, whole-grain products, beans, prunes, etc.
Fluoride	Females: ≥19 y: 3mg ^Γ^ Males: ≥19 y: 4 mg ^Γ^	Bone and teeth formation; prevents tooth decay [20].	Fish, teas, etc.
Iodine	≥19 y: 150 mcg ^	Part of thyroid hormone. [20]	Iodized salt, seafood, etc.
Iron	Females: 19–50 y:18 mg ^Δ,^^; ≥51y: 8 mg ^Δ,^^ Males: ≥19 y: 8 mg ^Δ,^^	Part of hemoglobin; energy metabolism [20].	Red meat, poultry, eggs, fruits, green vegetables, fortified bread, etc.
Manganese	Females: ≥19 y: 1.8 mg ^Γ^ Males: ≥19 y: 2.3 mg ^Γ^	Part of several enzymes [20].	Nuts, legumes, whole grains, tea, etc.
Selenium	≥19 y: 55 mcg ^	Serves as an antioxidant [20].	Organ meat, seafood, walnuts, etc.
Zinc	Females: ≥19 y: 8 mg ^Δ,^^ Males: ≥19 y: 11 mg ^Δ,^^	Enzymes; protein and genetic material synthesis; taste perception, wound healing, normal fetal development, production of sperm, normal growth and sexual maturation, immune system health. [20]	Meat, shellfish, legumes, whole grains, etc.

Retinol Activity Equivalents (RAE); International Units (IU); Alpha-Tocopherol (AT); ^Δ^ 2020–2025 United States Department of Agriculture Dietary Guidelines for Americans; ^ Recommended Daily Allowance; ^Γ^ Adequate Intake; ^δ^ Chronic Disease Risk Reduction level; ^ϑ^ Paralyzed Veterans of America Clinical Practice Guidelines on the Identification and Management of Cardiometabolic Risk After Spinal Cord Injury; ^α^ American Heart Association.

**Table 3:** MyPlate’s Fruit Group with Examples for Each Fruit Form and Recommended Intakes.

Fruit Form	Cup Equivalent	Examples
Fresh	1 piece of whole fruit or 1 cup of cut fruit	Apples, grapes, clementine, banana, or any type of berry (blackberries, blueberries, raspberries, strawberries), etc.
Frozen	½ cup of thawed fruit or 1 cup of frozen fruit	Bananas, apples, pineapple, berries, sapote, soursop, papaya, etc.
Canned	1 cup of canned fruit	Peaches, pears, apricots, pineapples, etc.
Dried	½ cup of dried fruit	Cranberries, raisins, apples, mango, pineapple, blueberries, dates, prunes, apricots, etc.

Examples of fruits that fall under MyPlate’s Fruit Group with examples for fresh, frozen, canned, and dried forms based on the 2020–2025 United States Department of Agriculture Dietary Guidelines for Americans [18]. Fruit should be canned in its own juice rather than a heavy syrup. Dried fruit ideally should not have added sugar.

**Table 4:** MyPlate’s Vegetable Group with Examples for Each Vegetable Subcategory and Recommended Intakes.

Vegetable Subgroup	USDA’s Recommended Intake	Examples
Dark green vegetables	1.5 cup equivalent/week	Broccoli, bok choy, chard, collards, kale, romaine lettuce, spinach, turnip greens and watercress, etc.
Red and orange vegetables	5.5 cup equivalents/week	Carrots, red/orange bell peppers, sweet potatoes, tomatoes, and winter squash, etc.
Beans, peas, and lentils	1.5 cup equivalents/week	Black beans, black-eyed peas, chickpeas (garbanzo beans), edamame, kidney beans, lentils, lima beans, pinto beans, split peas, etc.
Starchy vegetables	5 cup equivalents/week	Corn, cassava, green peas, white potato, taro root, water chestnuts, yam, yucca, etc.
Other vegetables	4 cup equivalents/week	Asparagus, avocado, beets, Brussels sprouts, cabbage, cauliflower, celery, cucumber, eggplant, green/string beans, iceberg lettuce, mushrooms, okra, onions, radish, seaweed, etc.

Examples of vegetables that fall under each subcategory and their respective recommended intakes of the Vegetable Group based on the 2020–2025 United States Department of Agriculture Dietary Guidelines for Americans [18]. Recommended intakes of the Vegetable Group are measured in cup equivalents per week.

**Table 5:** MyPlate’s Grain Group and Examples of Whole/Refined Grains with Recommended Intakes Based.

Grains	Examples	Recommended Intake
Whole-Grains	Whole wheat flour, bulgur wheat (cracked wheat), oatmeal, amaranth, barley, millet, popcorn, brown rice, quinoa, dark rye, wild rice, etc.	At least 3-ounce equivalents per day (maximum 6-ounce equivalents of grains per day)
Refined-Grains	White flour, corn grits, white bread, white pasta, white rice, cream of rice, cream of wheat, etc.	Less than 3-ounce equivalents per day (maximum 6-ounce equivalents of grains per day)

United States Department of Agriculture 2020–2025 Dietary Guidelines for Americans recommendation of grains is 6-ounce equivalents per day, with at least 3-ounce equivalents from whole-grains [18]. Examples of an ounce equivalent are ½ cup cooked rice, pasta, or cereal; 1-ounce of dry pasta or rice, 1 medium slice of bread, tortilla, flatbread, or 1 cup of flaked cereal [18].

**Table 6:** MyPlate’s Protein Group with Sources, Examples, and Recommended Intake.

Protein Source	Recommended Intake	Examples
Meats/Poultry/Eggs	26-ounce equivalents/week	Meats: Beef, goat, lamb, pork and game meat (e.g., moose, elk, deer, etc.) Poultry: Chicken, Cornish hen, duck, goose, turkey, game birds (e.g., ostrich, pheasant, quail, etc.) Eggs: Chicken eggs or any other birds’ eggs
Seafood	8-ounce equivalents/week	Salmon, sardines, tuna, shrimp, tilapia, anchovy, black sea bass, catfish, clams, cod, crab, flounder, haddock, oyster, squid, etc.
Nuts/Seeds/Soy Products	5-ounce equivalents/week	Nuts and seeds: All nuts (tree nuts and peanuts), nut butters, seeds (e.g., chia, flax, pumpkin, sesame and sunflower, etc.). Soy products: Tofu, tempeh, and products made from soy flour, soy protein isolate, soy concentrate, etc.

United States Department of Agriculture 2020–2025 Dietary Guidelines for Americans recommendation for protein intake is 5.5-ounce equivalents/day. An ounce-equivalent of protein food is 1 ounce of lean meat/poultry or seafood, 1 egg, ¼ cup of cooked beans or tofu, 1 tbsp of nut/seed butter, and ½ ounce of nuts/seeds [18]. Meat and poultry should be lean or low-fat. Highly processed meats (e.g., hot dogs, sausage, ham, luncheon meats, etc.) should be avoided. Nuts should be unsalted or lightly salted to avoid excessive sodium intake. Green/string beans or green peas are not included in the beans, peas, or lentils subgroup.

**Table 7:** MyPlate’s Dairy Group with Types, Examples, and Recommended Intake.

Type	Form	Cup-Equivalent	Examples
Milk	Fluid	1 cup	Dairy milk, lactose-free or reduced milk, fortified soy milk, buttermilk, dairy desserts
	Dry	1 cup (reconstituted in water)	
	Evaporated	½ cup	
Yogurt	Yogurt	1 cup	Yogurt, Greek yogurt, kefir, frozen yogurt
	Fortified soy-yogurt	1 cup	Soy-yogurt, soy desserts
Cheese	Natural cheese	1½ ounces	Brie, cheddar, cottage cheese, Colby, edam, feta, fontina, goat, gouda, gruyere, mozzarella, muenster, parmesan, provolone, ricotta, Swiss, etc.
	Processed cheese	2 ounces	American cheese

United States Department Agriculture 2020–2025 Dietary Guidelines for Americans recommendations for the Dairy Group are 3-cup equivalents per day for adults [18,46]. Fat-free or reduced-fat options are recommended. Milk alternatives (almond, rice, oat, etc.) or dairy-derived products with low calcium content (cream, sour cream, cream cheese) are not included in the Dairy Group.

## Supplementary Materials

The following supporting information can be downloaded at: https://www.mdpi.com/article/10.3390/jpm12122029/s1, Nutrition education to reduce metabolic disfunction for spinal cord injury: A module-based nutrition education guide for healthcare providers and consumers.
