# Supplementary material for: Nutrition Education to Reduce Metabolic Dysfunction for Spinal Cord Injury: A Module-Based Nutrition Education Guide for Healthcare Providers and Consumers
Source: J Pers Med. 2022 Dec 7;12(12):2029. doi: 10.3390/jpm12122029 (PMC9786330; doi:10.3390/jpm12122029)
Supplement: Supplementary file 1 [file jpm-12-02029-s001.zip › jpm-1945797 SM.pdf]

NUTRITION EDUCATION TO REDUCE METABOLIC DYSFUNCTION FOR SPINAL CORD INJURY: A MODULE-BASED NUTRITION EDUCATION GUIDE FOR HEALTHCARE PROVIDERS AND CONSUMERS

Alicia Sneij, Ph.D., M.S., RDN,[1,2] Gary J. Farkas, Ph.D.,[1,2] Marisa R. Carino Mason,[1] David R. Gater, M.D., Ph.D., M.S.[1,2,3,4]

1. Department of Physical Medicine and Rehabilitation, University of Miami Miller School of Medicine
2. Christine E. Lynn Rehabilitation Center for the Miami Project to Cure Paralysis
3. The Miami Project to Cure Paralysis, University of Miami Miller School of Medicine
4. South Florida Spinal Cord Injury Model System, University of Miami Miller School of Medicine,

October 17, 2022

MODULE 1 - MYPLATE: A GUIDE TO HEALTHY EATING

MyPlate is based on the 2020-2025 Dietary Guidelines for Americans. It serves as the model for healthy meals for Americans ages 2 and over and helps us make better food choices.

KEY RECOMMENDATIONS OF THE DIETARY GUIDELINES FOR AMERICANS:

- ✓ **Balancing calories**
  - Enjoy your food but reduce oversized portions.
- ✓ **Increase nutritionally dense foods**
  - Make half your plate fruits and vegetables.
  - Make ¼ of your plate whole grains such as brown rice or couscous.
  - Switch to fat-free or low-fat (1%) milk.
- ✓ **Focus on the five MyPlate food groups.**
  - Fruits, vegetables, grains, protein, and dairy (glass of skim milk or cup of non-fat yogurt).
- ✓ **Be conscientious of your sodium intake.**
  - In foods like soup, other canned products, and frozen meals, ask for the ones with the lowest sodium or ask for special low sodium soup alternatives.
- ✓ **Drink water instead of sugary drinks.**

EAT **MORE** OF THIS:

- Vegetables
- Fruits
- Whole grain
- Lean protein
- Seafood
- Low fat dairy
- Healthy oils (olive or avocado)

EAT **LESS** OF THIS:

- Sugar
- Salt
- Fast food
- Refined grains
- Saturated and trans fats

Source: U.S. Department of Agriculture (www.myplate.gov)

MODULE 2 – THE FRUIT GROUP

What foods are in the Fruit Group?

Any fruit or 100% fruit juice counts as part of the Fruit Group. Fruits may be fresh, canned, frozen, or dried, and may be whole, cut-up, or pureed (mashed), like applesauce.

How much fruit is needed daily?

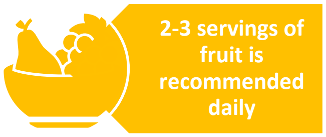

Two to three servings of fruit each day is recommended. If you think about it, the variety of fruit is big and it can be eaten in so many forms that it's pretty easy to eat 2-3 servings a day. Look at the various forms fruit can be consumed and think about ways you may be able to creatively add more fruit to your daily diet.

Fresh fruit

- For example: apple, grapes, an easy to peel clementine, any type of berry or a banana can be part of your meal or a nutrient-rich snack. Added to cereal or eaten alone, whole fruit is higher in fiber than juice.
- One piece of whole fruit or a cup of fresh fruit = 1 serving

Juices

- Orange, grapefruit, cranberry, cherry, or apple juice are good options. 1 cup = one serving of fruit. Juice has almost no fiber and may have added sugars. Read the nutrition facts label and make sure you're drinking pure fruit juice instead of a fruit drink.

Canned fruits

- Packed in their own juice (not heavy syrup), canned fruits are a great alternative.
- One cup = one serving of fruit
- Canned peaches, pears, apricots, and pineapples are not found as fresh foods in grocery stores year-round so eating them canned makes it easy to eat them anytime.

Frozen fruit

- Frozen fruit is typically high in nutrients because its flash frozen right upon harvest.
- Choose strawberries, peaches, or mixed fruit or berries. Add to milk or yogurt and blend into a fruit smoothie. If you thaw frozen fruit you can eat it as a fruit sauce, add it to oatmeal, or mix in with yogurt.
- A ½ cup of *thawed* frozen fruit or a cup of frozen fruit = one serving of fruit.

Dried fruit

- Add dried cranberries (craisins) or raisins to oatmeal or other cereals.
- Almost every grocery store carries a variety of dried fruit that includes dried apples, mango, pineapple, blueberries, dates, prunes (dried plums), and apricots.
- These are excellent sources of vitamin A and C – like most fruits!
- A half cup of any dried fruit = one serving of fruit.
- Always check the nutrition facts label to be sure.

Module 3 – The Vegetable Group

What foods are in the Vegetable Group?

- ✓ Any vegetable or 100% vegetable juice
- ✓ Raw or cooked vegetables
- ✓ Fresh, frozen, canned, or dried/dehydrated vegetables
- ✓ Whole, cut-up, or mashed vegetables

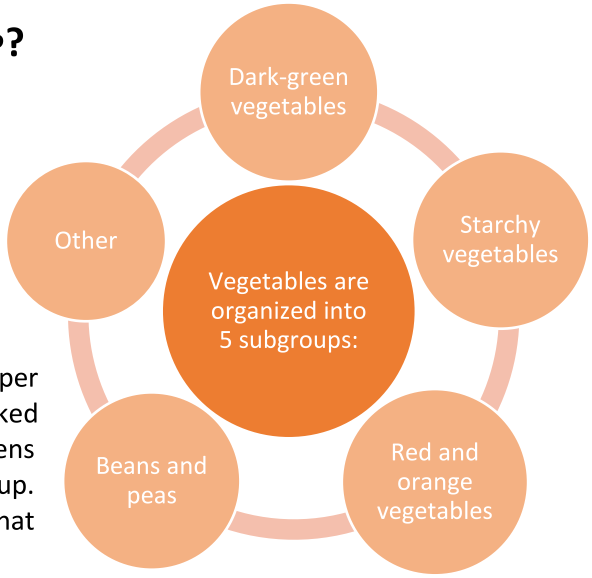

How many vegetables are needed?

The total recommended amount of vegetables needed per day is about 2-3 cups. In general, 1 cup of raw or cooked vegetables or vegetable juice, or 2 cups of raw leafy greens can be considered as 1 cup from the vegetable group. Common vegetables listed below shows specific amounts that are equivalent to 1 cup of vegetables.

Broccoli
- 1 cup raw or cooked

Carrots
- 1 cup raw or cooked
- Raw carrot sticks from 2 medium carrots

Bell peppers
- 1 cup raw or cooked

Spinach or other leafy greens
- 2 cups raw or 1 cup cooked, chopped, or canned

Sweet potato
- 1 large baked or 1 cup cooked (mashed)

Corn, peas, white potato, beans
- For example, 1 cup of garbanzo beans, lentils, white or kidney beans

Vegetables can be eaten at breakfast, lunch, and dinner or as snacks. It takes conscientious effort to meet vegetable intake goals so planning ahead to ensure vegetables are in your refrigerator and pantry is a must! Planning to eat a salad every day is an automatic winner for coming close to your goal and provides a great opportunity to include 4 or more vegetables at once.

Try out this Salad Recipe, which equals to 1 serving of vegetables for the day!

1 cup of raw spinach leaves + 1 cup of raw chopped lettuce
½ cup chopped raw carrot + ½ cup raw chopped tomato

Module 4 – The Grain Group

What foods are in the Grains Group?

Grains are divided into 2 subgroups, Whole Grains and Refined Grains. ***Whole grains*** contain the entire kernel of the grain— the bran, germ, and endosperm. Examples of whole grains include whole-wheat flour, bulgur (cracked wheat), oatmeal, whole cornmeal, and brown rice. ***Refined grains*** have been milled, a type of processing that removes the bran and germ. Essentially removing the nutritious fiber and some other nutrients. This is done to give grains a finer texture and make them last longer on your pantry shelve. *Some examples of refined grain products* are white flour, white bread, white rice and processed cereals.

Any food made from wheat, rice, oats, cornmeal, barley, or another cereal grain is included in the grain group.

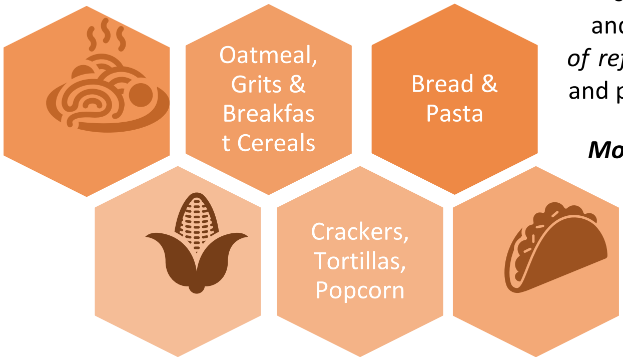

Most refined grains are enriched. This means certain B vitamins (thiamin, riboflavin, niacin, folic acid) and iron are added back after processing. Fiber is not added back to enriched grains. ***Check the ingredient list on refined grain products to make sure that the word "enriched" is included in the grain name.*** Some food products are made from mixtures of whole grains and refined grains.

How many grain foods are needed daily?

6-11 SERVINGS OF GRAINS ARE RECOMMENDED DAILY WITH 50% FROM WHOLE GRAINS

The amount of grains needed per day ranges from 6-11 servings depending on your energy needs. Most people eat enough grains, but few are whole grains. At least half of all the grains eaten should be whole grains. Ask for whole grain bread and cereals when someone asks. Even pizza crust can be made with whole grain flour!

In general, 1 slice of bread, 1 cup of ready-to-eat cereal, or ½ cup of cooked rice, cooked pasta, or cooked cereal (such as oatmeal) can be considered as 1 serving from the grain group. Other foods frequently placed in the grain group, due to their high starch content, include potatoes, corn, and peas. A serving of these starchy vegetables is ½ cup.

What is a serving of grain?

Foods in the grain group must be eaten in small portion sizes - smaller than the average person expects! This list includes common foods and the servings of grains associated with it.

1 whole English muffin = 2 servings
2 cups of dry cereal (e.g. Frosted Flakes) = 4 servings
2 pieces of toast = 2 servings
1/4 cup Grapenuts cereal = 1 serving
2 cups of cooked pasta = 4 servings
1 cup white or brown rice = 2 servings
5 Triscuit crackers = 1 serving

Reading the Nutrition Facts Label on any cereal box or bread package will give you more accurate information specific to the product you want to eat. Although foods in the grain group provide iron, B vitamins, and if "whole grain", fiber, portion size eaten in this food group is challenging for many. The more you understand what grains you eat, the greater the opportunity to shift to whole grains or just reduce processed grains if you think you eat more than you need.

MODULE 5 – THE PROTEIN GROUP

- ***Protein provides 4 calories/gram and is an essential nutrient we can't live without.***
- ***It is a KEY nutrient for skin and muscle health.***

WHAT DOES IT DO?

1. **Build antibodies for fighting infections, colds, and the flu.**
2. **Maintain and build muscle.**
3. **Help keep us satiated or feeling full longer after a meal.**
4. **Repair and build healthy skin tissue.**

HOW MUCH DO I NEED?

Getting about half of your body weight in grams of protein per day is an easy rule of thumb. For example, if you weigh 150 pounds, you should aim for about 75 grams of protein per day.

- **Getting enough protein along with key nutrients such as Vitamin A and C can help prevent or treat pressure ulcers. Getting enough protein can also help prevent muscle loss.**

WHERE CAN I GET IT?

CHOOSE LEAN (LOW-FAT OR NON-FAT) PROTEIN FOODS WHENEVER POSSIBLE TO STAY WITHIN YOUR CALORIE AND FAT GRAM GOALS.

Chicken, Turkey, and Duck

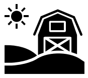

All milk (skim), yogurt, cheese and cottage cheese, kefir

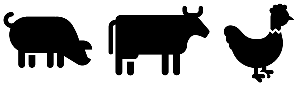

Pork, Beef, and Bison

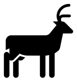

Hummus made from beans

Beans: black, lentil, garbanzo, pinto, or kidney

Tofu, bean burger, nuts of all sources

Game meat such as Deer, Rabbit, or Elk

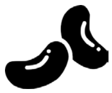

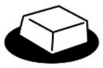

Tahini – it's sesame butter and typically used in making hummus.

Module 6 – The Meat or Meat Alternatives Group
(Protein Foods)

All foods made from meat, poultry, seafood, beans and peas, eggs, processed soy products, nuts, and seeds are considered part of the Meat or meat alternatives group or called the Protein Foods Group. Beans and peas are also part of the Vegetable Group.

There is a wide variety of protein foods that help build all tissues and new cells even though most people associate these foods with only muscle growth. ***Protein helps all body systems and protein rich foods provide many vitamins and minerals.***

How much food from the Protein Foods Group is needed daily?

The amount of food from the Protein Foods Group you need *depends on many factors such as age, weight, and physical activity level*. Most people eat adequate protein, but need to make leaner, low fat choices from a wider variety of foods.

How Much Protein Do You Need?

Most women over the age of 19 need 5-6 oz of protein/day and men over 19 need 6-7 oz of protein/day.

Women 19+ years old	5-6 oz of protein/day
Men 19+ years old	6-7 oz of protein/day

Listed are examples of what constitutes a one serving of protein.	1 ounce of meat, poultry or fish
	1/4 cup of cooked beans
	1 egg (3 egg whites or 3 egg yolks)
	1 tablespoon of peanut butter
	1/2 ounce of nuts or seeds

Getting more protein from vegetable sources is getting widespread attention as having many health advantages. Low in saturated fat beans and peas are unique sources of protein.

Protein rich beans and peas

Beans and peas are part of the meat alternative/protein food group. They include kidney beans, pinto beans, black beans, lima beans, black-eyed peas, garbanzo beans (chickpeas), split peas and lentils. Similar to meats, poultry, and fish, beans are rich in iron and zinc. Many people consider beans and peas as vegetarian alternatives for meat.

Because of their high nutrient content, consuming beans and peas is recommended for everyone, including people who also eat meat, poultry, and fish regularly. One full cup of beans is the equivalent of 4 oz of meat.

Reminders

- Green peas or green string beans are not part of the meat alternative/protein group. They remain in the vegetable group.
- Hummus, typically made with garbonzo beans (chickpeas) is an excellent protein source. Bean soup or lentil soup, canned or fresh, are excellent meat alternatives.

MODULE 7 - THE DAIRY GROUP

WHAT FOODS ARE INCLUDED IN THE DAIRY GROUP?

All fluid milk products and many foods made from milk that retain their calcium content are considered part of this food group. Calcium-fortified soymilk (soy beverage) is also part of the Dairy Group. Foods made from milk that have little to no calcium, such as cream cheese, sour cream, cream, and butter are not.

HOW MUCH FOOD FROM THE DAIRY GROUP IS NEEDED DAILY?

The amount of food from the Dairy Group you need to eat depends on age. **Recommended daily amounts for adults are three servings or 3 cup-equivalents per day**.

WHAT COUNTS AS A CUP-EQUIVALENT IN THE DAIRY GROUP?

In general, 1 cup of milk, yogurt, or fortified soymilk, 1 ½ ounces of natural cheese, or 2 ounces of processed cheese can be considered as 1 cup-equivalent from the Dairy Group. When choosing dairy**, fat-free and low-fat dairy are best because they're lower in calories.** The table below lists specific amounts that count as 1 cup-equivalent in the Dairy Group toward your daily recommended intake.

	Amount that Counts as 1 Cup in the Dairy Group
**Milk**	1 cup milk (or ½ pint)
	1/2 cup evaporated milk
	1 cup of calcium fortified soy milk (or ½ pint)
**Yogurt**	1 cup of yogurt (dairy or fortified soy)
	1 cup of Greek yogurt
**Cheese**	1 ½ oz of hard cheese (cheddar, mozzarella, Swiss, Parmesan)
	1/3 cup of shredded cheese
	2 oz of processed cheese (American)
	½ cup of ricotta cheese
	2 cups of cottage cheese
	2 oz Queso fresco
	2 slices of Queso blanco

Module 8 – Fat

Fat is an energy-rich nutrient (9 calories/gram) that is found in many foods. Fat makes food taste creamy, smooth, and delicious…which are reasons we like it so much! In meats, fat helps maintain moisture, reducing the "toughness" and causing meat to taste "juicy." But unfortunately, excess fat in your diet may cause unwanted weight gain.

Some people are afraid to eat fat due to the higher calories. ***But several types of fats are actually healthy for you!*** Below is a list of function that fat performs in the body:

- Makes hormones and stores Vitamins A, D, E, and K.
- Maintains the health of your brain and nervous system (60% of your bain is made of fat, so you need good fats to support optimal brain function).
- Lubricates joints, maintains healthy skin and improves wound healing.
- Prevents cardiovascular disease decreases inflammation.

There are several different types of fat. It's important to understand the difference between fats that are less healthy and fats that are better for you, so you can make healthier choices.

UNSATURATED FATS

The unsaturated fats, which include both monounsaturated and polyunsaturated fats, are often called the "good" or "healthy" fats. These fats include the essential fatty acids discussed in the following Module.

Monounsaturated fats more than polyunsaturated fats can ***lower the LDL, or "bad" cholesterol in your body, thus reducing the risk of heart disease***.

SATURATED FATS

Another type of fat is called saturated fat. This type of fat has been associated with ***elevating cholesterol as well as causing increased inflammation***. It's very hard to avoid saturated fat because it's naturally found in meats, cheese, and other food that comes from animals. Milk for example, contains saturated fat if it's not "skim" milk.

MODULE 9 – ESSENTIAL FATTY ACIDS

- **Healthy fats** provide us with essential fatty acids, such as omega 3 and 6 fatty acids.

- Fatty acids that are essential means that they **cannot** be made in the body, while non-essential fatty acids can be!

- Non-essential fatty acids are regular fats in food BUT are produced by the body, which is why they are called non-essential.
    - Most people have very little trouble getting enough non-essential fatty acids into their diet. *In fact, reducing intake of these fats should be a goal for achieving optimal health.*

Omega 3

- Reduce inflammation, prevent cardiovascular disease, maintain healthy cholesterol levels and reduce obesity risk.
- Supports brain function and the immune system as well.
- Sources of omega-3 include salmon, mackerel, chia, flax, and walnuts.
- Can also be taken as a supplement, but always check with your doctor first before considering its use.

Omega 6

- Supports normal health and development as well as provides good heart health benefits.
- Can be found in vegetable oils such as corn, soybean, safflower, and sunflower oils, as well as nuts.

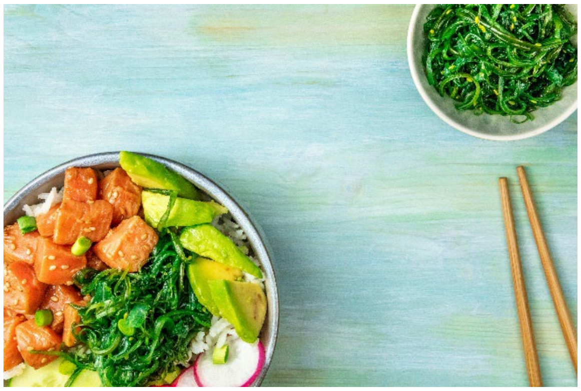

Module 10 –Ways to Eat Less Fat and Fewer Calories

1. Eat high-fat, high-calorie foods ***less often***.

- For example, avoid eating french fries every day. Instead, have them only once a week. (That's about 132 fewer grams of fat per week!)
- Cutting back even a little on the amount you eat can make a big difference.
- There are many lower fat, lower-calorie items in the grocery stores. For example, low-fat salad dressing, skim milk, low-fat yogurt and cheese, and lean cuts of meat.

2. Eat ***smaller*** portion sizes of high-fat, high-calorie foods.

3. Eat ***lower-fat, lower-calorie*** foods instead.

4. Check the label for calorie density per serving size!

- For example, 1/2 cup of nonfat frozen yogurt equals 100 calories, compared to 1/2 cup of regular ice cream 143 calories, which equals about a 10-12% difference in fat content.

WARNING: Low-fat or fat-free products still contain calories. Be careful about *how much* you eat. In fact, some low-fat or fat-free products are very high in calories because they are loaded with sugar.

Small changes in the portion sizes or food types can go a long way to help eat less fat and fewer calories. The following graphic lists a few examples of easy switches that you can implement into your daily eating habits.

Potato chips, 1-ounce bag<br>150 cal, 10g fat	→	Pretzels, 1-ounce bag<br>100 cal, 0g fat
Regular margarine, 1 tsp<br>35 cal, 4g fat	→	Light margarine, 1 tsp<br>20 cal, 2g fat
Sour cream, 2 tbs<br>50 cal, 5g fat	→	Salsa, 2 tbs<br>15 cal, 0g fat
Roast beef (chuck), untrimmed 3oz<br>310 cal, 24g fat	→	Roast beef (top round), rimmed, 3oz<br>160 cal, 5g fat
4oz breaded, fried chicken breast with skin<br>315 cal, 17g fat	→	4 oz roasted chicken no skin<br>175 cal, 4.5g fat

MODULE 11 – CARBOHYDRATES

Carbohydrates provide your body with the energy (4 calories/gram) it needs for your tissues, muscles, nerves, and brain to function properly. No matter what kind of carbohydrates you eat, they are all eventually broken down in your body to glucose. Glucose is the simplest of all sugars and what ultimately provides your body with energy. All sugar – no matter what kind it is will become glucose in the body for use as energy.

Plant foods are the source of almost all carbohydrates. They are found in mineral-rich and vitamin-rich vegetables, fruits, grains, and legumes (beans & peas). The only animal foods that contain carbohydrates are dairy products in the form of the milk sugar called lactose.

Some people avoid eating carbohydrates because they believe they contribute to weight gain. **The reality is that carbohydrates can be easily "over-eaten."** For example, when pasta is eaten, people eat an entire **PLATE** of it rather than a **PORTION** of it. Other carb-rich foods that are easily overeaten are mashed potatoes, potato chips (also high in fat), cookies, cake, bread, pancakes, and the list could go on and on. In the world of nutrition there are some carbs that are better for you to eat than others. **Usually, the lower calorie carb sources are the best and therefore called the "Good Carbohydrates."**

GOOD CARBOHYDRATES – WHOLE VEGETABLES, FRUITS, GRAINS, AND BEANS

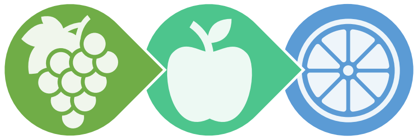

These foods contain ***fiber, vitamins, minerals,*** and other chemicals called ***"anti-oxidants"*** to help give you long-lasting energy, control your blood sugar levels, and maintain your health and ideal body weight. Eating the right amount of these carbohydrates should be your goal.

BAD CARBOHYDRATES – REFINED SUGARS FOUND IN SODA, COOKIES & CANDY, AS WELL AS PROCESSED GRAINS, SUCH AS WHITE BREAD & WHITE PASTA

These may also contribute to weight gain because they are easily overeaten. They also are typically very low in fiber, vitamins, and minerals. It is important to make an effort to reduce these and focus on the nutrient rich variety of carbohydrates.

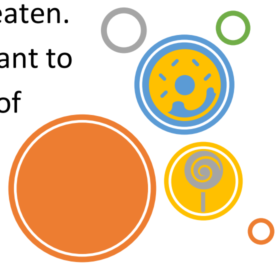

Module 12 –Fiber

Fiber is found only in plant foods such as fruits, vegetables, and whole grains.

Fiber has many health benefits such as:

- ✓ Helps to control weight by stimulating you to feel full
- ✓ Low-calorie intake
- ✓ Improves digestion and gastrointestinal (GI) health
- ✓ Keeps cholesterol levels healthy
- ✓ Helps control blood sugar levels
- ✓ May decrease risk of some cancer

There are two types of fiber:

1. Soluble fiber	2. Insoluble fiber
• **Helps lower cholesterol and controls blood glucose**	• **Adds bulk to stools by soaking up water, which helps keep the GI track moving things through.**

Men: ~30 grams/day

Women: ~25 grams/day

Getting your fiber intake just right can be a challenge when you have impaired mobility. Too little may lead to constipation or bowel impactions. Too much can lead to painful bloating, excess gas, and needing to use the bathroom more often.

The USDA states that a person should try to eat 14 grams of fiber for every 1000 calories they eat/day. It is recommended that individuals consume 22-34 grams of fiber/day (age/sex dependent).

Tips: When increasing fiber in your diet, start slowly. Start with about 15 grams of fiber each day, then slowly increase up to 30 grams per day if tolerated. Your GI tract takes time to adjust to more fiber. As you increase your fiber intake, be sure to drink plenty of water. Too much fiber without enough water may cause constipation.

Where can I get more fiber?

- Fruits and Vegetables
- Whole grain breads and cereals
- Brown Rice
- Whole Grain Bulgur Wheat
- Nuts and Seeds
- Dried fruits
- Whole wheat pasta and crackers
- Oats or barley
- Peelings on fruits and veggies!

MODULE 13 – PLANNING A HEALTHY BREAKFAST

Start your day off right by eating foods that serve a purpose by giving you energy, protein, and a variety of vitamins and minerals. **MyPlate** recommendations can provide a great guideline for all meals, but a nutritious breakfast sets the stage.

VEGETABLES

Out of all the food groups, veggies are the toughest to fit into breakfast. Here are a few reasonable suggestions:

- ✓ Add vegetables to egg dishes (spinach, onions, garlic, mushrooms, red or green pepper).
- ✓ Drink tomato, vegetable (V8) or carrot juice.
- ✓ Blend vegetables such as kale, spinach or other greens into a smoothie.

FRUITS: AN EASY FOOD TO EAT FRESH, CANNED, DRIED, OR FROZEN!

- ✓ **Fresh fruit** like grapes, clementine, berries or bananas can be the start of your high fiber breakfast. Added to cereal or eaten alone, whole fruit is higher in fiber than juice.

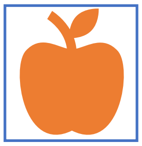

- ✓ Orange, grapefruit, cranberry, cherry or apple juice are great options, but may have added sugars and contain almost zero fiber. **Read the nutrition facts label and make sure you are drinking *pure* fruit juice as opposed to a fruit drink.**

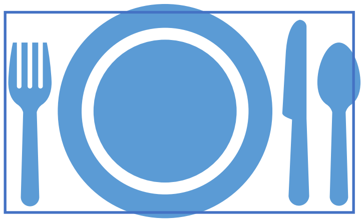

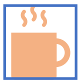

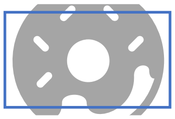

- ✓ **Canned fruits** packed in their own juice (without heavy syrup). A typical serving size is 1/2 - 3/4 cup.

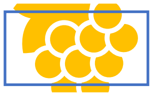

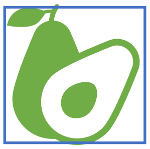

- ✓ **Frozen fruit** is typically high in nutrients because it is flash frozen right upon harvest. Choose strawberries, peaches, or mixed fruit to add to milk or yogurt, and blend into a fruit smoothie.
- ✓ Add **dried fruit** such as dried cranberries (Craisins) or raisins to oatmeal or other cereals. Almost every grocery store carries a variety of dried fruits that include dried apples, mango, pineapple, blueberries, and apricots. Some of these, such as mango and apricots, are almost easier to find in the dried form. These are excellent sources of vitamin A and C - like most fruits!

Grains: This is the food group most easily over-eaten! Be mindful of portion sizes.

Choose oatmeal or cereals that are low in added sugar and high in fiber. Include whole grain toast, an English muffin, bagel, or tortilla for a breakfast burrito.

- ✓ **Eat less often**: "frosted" or sweetened cereals, cereals with nuts or coconut which are higher in calories; high fat croissants, biscuits, and most muffins. These foods are typically higher in calories and fat. If chosen occasionally, be mindful of portion size and if possible, review the Nutrition Facts label for portion size, fat, and calories per serving.

Protein

To get protein in at breakfast you need to include food from the dairy or meat/egg food groups. If you are not eating animal-based foods (vegan or vegetarian) then your protein can adequately come from grains and vegetables, like soy products and nuts.

- ✓ Include eggs, egg substitutes, yogurt, milk, or cheese
- ✓ **Vegan or Vegetarian options include** soy products like soy milk, cheese, or yogurt; peanut or almond butter on toast; or opt for a tortilla with refried beans spread on it.
- ✓ Eat less often: bacon or sausage.

Dairy: This could come into your breakfast as your protein source!

- ✓ Choose skim, 1%, or soy milk, nonfat or low-fat and low-sugar yogurt or cheese. Almond or coconut milk are not part of the dairy group.
- ✓ **Tip**: all varieties of milk contain 8 grams of protein. By drinking skim or 1% milk you save a lot of fat but still get the quality protein. Almond or coconut milks are very low sources of protein.
- ✓ **At breakfast:** try to use these ingredients or foods sparingly: butter, sugar, honey, regular syrup, cream cheese, pastries, coffeecake, doughnuts, and sugar-rich coffee drinks.

MODULE 14 – BUILDING A "LIGHT" MEAL

Make one meal each day a "light" meal.

Have a simple, light lunch if you're planning to go out to dinner. Or make dinner a light meal if you have eaten a large lunch. **MyPlate** recommendations serve as the model for these suggestions.

- ✓ Sandwich, carrot sticks, fruit, milk.
- ✓ Salad, tuna, whole grain crackers.
- ✓ Pasta, veggies, beans.
- ✓ Brown rice, stir-fried veggies, chicken.
- ✓ Burrito, salad, fruit.
- ✓ Frozen entree, salad, fruit, milk.

ASK FOR HEALTHY FOODS!

With the following foods in the fridge, freezer, or pantry, a quick lighter meal can be made for you in no time.

Grains	Fruits & Vegetables	Dairy	Protein
• Low-fat breads (whole grain bread, bagels, English muffins, plain rolls, pita bread, tortillas)<br>• Low-fat crackers<br>• Cold or hot cereal<br>• Quick cooking brown rice<br>• Pasta	• Peeled carrots<br>• Prepared raw vegetables<br>• Salad greens<br>• Canned fruit (in water or juice)<br>• Canned tomatoes<br>• Frozen mixed vegetbles<br>• Vegetable soup (made with broth instead of cream)	• Skim or 1% milk<br>• Low-fat soy milk<br>• Nonfat or low-fat cheeses, fat free yogurts	• Canned, water-packed tuna, salmon, chicken<br>• Sliced turkey or chicken breast<br>• Sliced extra lean ham<br>• Canned beans (garbanzos, black beans, kidney or blackeye peas)<br>• Vegetarian refried beans

Module 15 – Satisfying Snacks

- Choose a healthy snack that matches the taste and texture you're looking for. But remember to watch the portion size. **Snacks should provide no more than 200 calories.**

- Portion-controlled options, such as fresh fruit, are always a good choice. But some other good choices include the following:

Fresh

- Fresh fruit (apple, pear, berries, banana, orange or tangerine)
- Raw vegetables (broccoli, carrots, cauliflower, green/red peppers, celery) with hummus
- Low-fat cottage cheese with fruit

Frozen

- Frozen grapes, bananas, berries
- Frozen fruit bars
- Lemon ice
- Frozen yogurt, nonfat Sherbet or sorbet

Other ideas

- Low-fat crackers (oyster, Melba, rice crackers, crispbread) with low-fat cheese or peanut butter
- Popcorn, air-popped or light
- Pretzels
- Baked tortilla chips and salsa (a vegetable!!)
- Rice cakes, popcorn cakes
- 100 calorie pack of nuts or trail mix
- Dried fruit (raisins, apricots)
- Chewy breads (English muffin, bagel, or slice of toast)
- Applesauce, unsweetened with cinnamon
- Low-fat or nonfat pudding
- Yogurt, light or nonfat Fruit Smoothie
- Whole grain graham crackers

Module 16 - Reading the Nutrition Facts Label

- ✓ Look at the Serving Size on the box, bag, or can that you might be eating from. How many servings are in there in the entire bag or box? What quantity makes up just one serving?
- ✓ Look at the Calories per Serving. ***The calories on the label are only for ONE serving.***
- ✓ Look at the Total Fat Grams per serving.
- ✓ What if you eat more than one serving? You'll be eating more calories and fat grams than are listed.

Consider this Nutrition Facts Label: Do you think this food would be considered healthy or nutritious?

**Nutrition Facts**	
8 servings per container	
**Serving size**	**2/3 cup (55g)**
**Amount per serving**	
**Calories**	**230**
	**% Daily Value***
**Total Fat** 8g	**10%**
Saturated Fat 1g	**5%**
Trans Fat 0g	
**Cholesterol** 0mg	**0%**
**Sodium** 160mg	**7%**
**Total Carbohydrate** 37g	**13%**
Dietary Fiber 4g	**14%**
Total Sugars 12g	
Includes 10g Added Sugars	**20%**
**Protein** 3g	
Vitamin D 2mcg	10%
Calcium 260mg	20%
Iron 8mg	45%
Potassium 240mg	6%

* The % Daily Value (DV) tells you how much a nutrient in a serving of food contributes to a daily diet. 2,000 calories a day is used for general nutrition advice.

Example food label sourced from www.fda.gov

This container has 8 servings of food, with each serving being 2/3 cup. Each value presented is only for 1 serving!

Each serving (2/3 cup of food) contains 230 calories. If you eat more than 1 serving, you have to multiply it by the # of calories for an accurate count!

The **protein is low** compared to the amount of **fat** per serving. As a result, you have the option of looking for a food that might be lower in fat and higher in protein per serving.

Also be mindful of the amount of **sodium** in each serving! Keep sodium intake below 2300mg each day, ideally under 1500mg.

Dietary fiber content is also something to keep an eye out for! Recommendations are 14g/1000 calories you consume.

You can also see the amount of **sugar** in each serving, including **added sugars**. Keep added sugar to <10% of total daily calories.

No one food is perfect regarding its nutrition profile, but the Nutrition Facts Label can serve as a resource that can assist you in making decisions about buying or eating a particular food. ***Look for nutrients that are good for you and try to keep fat low.***
